# Sorghum Starch and Protein Digestibility: Mechanisms, Modifications, and Health Implications

**DOI:** 10.3390/foods15101681

**Published:** 2026-05-12

**Authors:** Douglas Olson, Anbuhkani Muniandy, Lijia Zhu, Mohammad Zarei, Michael Schwarz, Scott Bean, Brennan Smith

**Affiliations:** 1 USDA-ARS-SRRC Food Processing and Sensory Quality, 1100 Allen Toussaint Blvd, New Orleans, LA 70124, USA; douglas.olson2@usda.gov; 2 Department of Animal, Veterinary, and Food Sciences, University of Idaho, 875 Perimeter Dr., Moscow, ID 83844, USA; 3 Virginia Seafood Agricultural Research and Extension Center, Virginia Polytechnic Institute and State University, Hampton, VA 23669, USA; 4 Center for Advanced Innovation in Agriculture (CAIA), Virginia Polytechnic Institute and State University, Hampton, VA 23669, USA; 5 USDA-ARS-CGAHR, Grain Quality and Structure Research Unit, 1515 College Ave, Manhattan, KS 66502, USA; scott.bean@usda.gov

**Keywords:** sorghum, digestibility, protein-starch interactions, resistant starch, food processing, functional foods

## Abstract

This review examines sorghum digestibility from molecular structure to clinical implications, focusing on compositional factors, processing methods, and health outcomes. We evaluate how sorghum’s unique protein–starch interactions influence digestibility and explore emerging technologies that can modulate these properties for targeted nutritional benefits. Cooked sorghum generally has lower digestibility than raw sorghum and other cereals due to heat-induced protein–starch cross-linking and the formation of disulfide bonds by sorghum proteins (kafirins), which restrict enzymatic access. Enzyme inhibitors in sorghum further reduce starch hydrolysis. This reduced digestibility may negatively impact malnourished individuals and those relying on sorghum as a dietary staple. However, it can be advantageous to individuals with diabetes by lowering postprandial blood glucose levels. Sorghum consumption may also beneficially influence the gut microbiome. Certain processing methods have been shown to significantly enhance digestibility while preserving beneficial bioactive compounds. Improving digestibility through these strategies may enhance sorghum’s value for vulnerable populations while maintaining its metabolic advantages. Balancing increased nutrient bioavailability with preservation of beneficial functional properties is critical for optimizing sorghum as a health-promoting grain across diverse populations.

## 1. Introduction to Sorghum

Sorghum (*Sorghum bicolor* L. *Moench*) is a commonly grown and valuable crop. Wild sorghum species were used between the eighth and fourth millennia B.C. in what is now the Sahara Desert in Egypt and Libya, and the first evidence of sorghum domestication goes back to the fourth millennium B.C. [1]. In 2024, sorghum was the fifth highest producing cereal crop in the world (production of 64,264,990.5 metric tons) after maize, wheat, rice, and barley and the fourth highest in the United States (production of 8,734,190 metric tons). The top five countries, in order, for sorghum production in 2024 were the United States, Nigeria, India, Sudan, and Mexico [2]. Beyond its nutritional importance, sorghum represents a critical crop for global food security and agricultural sustainability in the face of climate change. Its exceptional drought tolerance, lower water requirements compared to maize and rice, and ability to thrive in marginal soils make it particularly valuable for sustainable agriculture [3,4,5]. These environmental benefits, coupled with their nutritional profile, position sorghum as a strategically important grain for both human health and ecological sustainability. However, drought stress leads to an increased total protein content but decreased starch content and protein digestibility in sorghum [5]. Sorghum has complex and sometimes variable anatomical features, leading to different colors and classifications, and contains high concentrations of starch (amylose and amylopectin), fiber, and protein for supplying nutrients like other cereal grains.

**Figure 1:**
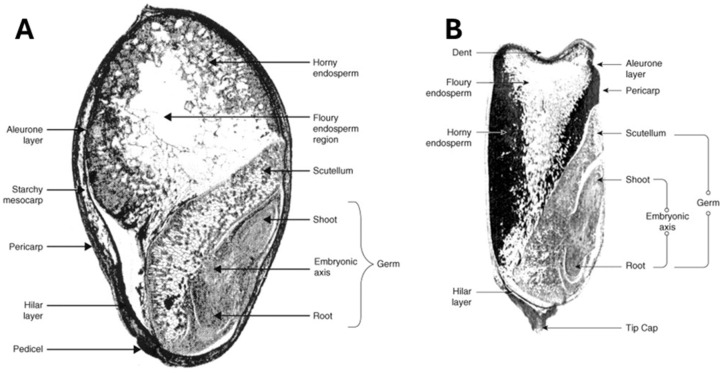
(**A**) A 22-fold magnification of an iodine stained longitudinal 10 μm bisection of a steeped grain sorghum kernel; (**B**) a 6-fold magnification of an iodine stained longitudinal 10 μm bisection of a steeped dent corn kernel [9]. (Figure 1A,B). Reprinted from Starch: Chemistry and Technology, Third Edition, S. R. Eckhoff and S. A. Watson, Corn and sorghum starches: Production, pp. 373–439, 2009, with permission from Elsevier.

The anatomical features of sorghum grain caryopsis consist of pericarp, endosperm, and germ, as shown in Figure 1A, and possibly a testa layer, and are similar to maize as shown in Figure 1B. The pericarp is the outer layer and is made up of three segments: the epicarp, mesocarp and endocarp [6]. The outermost layer, the epicarp, is usually covered with a thin waxy film. The mesocarp is the thickest component of the pericarp, with thickness varying with the genetic background of the sorghum variety. The endocarp is the innermost layer and consists of tube cells for moisture transportation. The testa layer is not present in all sorghum varieties due to variation in the genetics of the variety [7]. When present, the testa layer is located just underneath the endocarp. The presence of a testa indicates the presence of condensed tannins. The endosperm is the main storage organ for cereal grains including sorghum (Figure 1A). It contains the aleurone layer, peripheral vitreous endosperm, and floury endosperm. The aleurone layer is rich in vitamins, minerals and oils. The endosperm mainly consists of starch and cereal proteins. The germ contains the embryonic axis and scutellum and consists of lipids, proteins, amylolytic and proteolytic enzymes, and minerals [8].

Broad classification of colors of sorghum includes white, yellow, brown, red, and black [10]. Sorghum color is influenced by its phenolic compounds and determined by various genes as described by [10]. White sorghum contains low levels of phenolic compounds. Yellow sorghum contains flavanones. Brown sorghum contains high levels of condensed tannins, but red sorghum lacks tannins. Red sorghum and black sorghum are similar, and black sorghum contains high levels of phenolic compounds, particularly in the pericarp [10].

USDA Federal Grain Inspection Service (FGIS) has classified sorghum into four classes based on the color characteristics of the pericarps and/or testa of their kernels [11]. The four sorghum classes are “Sorghum”, “Tannin Sorghum”, “White Sorghum”, and “Mixed Sorghum”. “Sorghum” and “White Sorghum” lack a pigmented testa, while “Tannin Sorghum” has a pigmented testa. “Sorghum” contains less than 98.0% White sorghum and up to 3.0% Tannin sorghum, and the pericarp color can be white, yellow, pink, orange, red, or bronze. “White Sorghum” can contain up to 2.0% of sorghum from other classes, and the pericarp color is white or translucent. “Tannin Sorghum” can contain up to 10.0% of kernels that do not have a pigmented testa. Although the pericarp color of “Tannin Sorghum” is normally brown, it can also be white, yellow, pink, orange, red, or bronze. “Mixed Sorghum” does not meet the requirements for the other sorghum classes [11].

Nearly 40% of the world’s sorghum production is used for direct human consumption, often as a staple food, primarily in Africa and Asia [12]. Sorghum for human consumption is especially common in areas with limited food supplies and where hunger is a problem. Parts of the main meal, confectionary products, bakery products, porridge, beverages, breakfast products, and snack foods from sorghum are consumed in various African and Asian countries [13]. Granola with desirable sensory properties was prepared from a mix of micronized sorghum flakes and sorghum molasses as the main ingredients, and bars, cookies, rice cake-like products, and bite-size clusters can also be manufactured from this mix [14]. Sorghum grain is also used in the production of alcoholic beverages [3]. Since sorghum is naturally gluten-free, it has potential use as a main ingredient in gluten-free food products.

Sorghum-containing foods are not rapidly digestible by human enzymes, which could lead to malnutrition [15,16,17]. Malnutrition in children is common in sorghum-consuming areas. Therefore, it is extremely important to provide highly digestible starchy food to children. There are ways to manipulate sorghum starch and protein digestibility by modifying the processing methods to meet the dietary needs of these individuals [15]. Manipulating sorghum digestibility can benefit many populations, including patients suffering from celiac disease because of the suitability of the grain for gluten-free diets [12,15].

There are also innovative food ingredients and non-food uses for sorghum in addition to its use as a traditional human food. Sorghum is often used as animal feed in some countries including the United States, Brazil, and Australia and for commercial ethanol production [18]. Other products that can be made from sorghum include biopolymeric films and coatings, food colorants and edible cutlery, utility items, paper-based products [19], and porous sponge-like scaffolds in cultured meat production [20]. Sorghum can also be used in nanoparticle encapsulation of bioactive compounds [21].

Research interest in sorghum has increased from 2000 to 2020, as shown by the increased total number of publications per year. Key areas of research include genomics, climate resilience, biomass and bioenergy production, and bioactive components in sorghum. Recent research trends also highlight sorghum’s potential in sustainable food systems, with increasing focus on its role in addressing climate change challenges, nutritional security, and the development of novel value-added products [22]. Nutrition security depends upon starch and protein digestibility. A background in genetics, biochemistry, structure, and processing is necessary to fully understand sorghum starch and protein digestibility and is provided in this review paper. New information for several topics including genetics, protein structure and modification, processing techniques, and effect on the gut microbiome has become available within the last few years and is summarized in this review. This review paper provides insight into sorghum as a health-promoting food with an emphasis on analyzing the factors influencing its starch and protein digestibility, ways to improve its overall digestibility, and the effects on the gut microbiome.

## 2. Starch and Protein in Sorghum

### 2.1. Sorghum Starch

Starch is a polysaccharide consisting of D-glucose building blocks joined together to form either amylose or amylopectin. The average molecular weights of amylose from three varieties of sorghum were 6.4 × 10^6^, 7.1 × 10^6^, and 1.1 × 10^7^ g/mole as measured by size exclusion chromatography, and the average molecular weights of amylopectin from three varieties of sorghum were 2.9 × 10^8^, 3.0 × 10^8^, and 3.2 × 10^8^ g/mole as also measured by size exclusion chromatography [23]. The distinct types of chains within amylopectin include A-chains (short, unbranched chains that attach to B-chains without further branching), B-chains (intermediate length chains that connect with either A-chains or other B-chains), and a C-chain (a single C-chain with the only reducing end) [24]. Plants synthesize starch mainly within the endosperm in the form of starch granules to be used for energy storage, and starch is broken down to glucose by enzymes such as amylase and amylglucosidase. Starch content can be measured according to [25]. Resistant starch (RS) is the sum of starch and starch degradation products which are not absorbed within the small intestines of a healthy person and is considered a dietary fiber [26].

Contents of total starch, amylose, and amylopectin in sorghum are presented in Table 1. Starch containing very little to no amylose is referred to as waxy (wx) starch, and is produced in some plants, including waxy sorghum [27,28]. An amylose to amylopectin ratio at about 1:4 is the most common in plants. Starches with more than 40% amylose content are considered as high-amylose starch [29]. The ratio of amylose to amylopectin and the percentage of branched structures in amylopectin play a vital role in both the functionality and the nutritional properties of starch and starch-based products.

**Figure 2:**
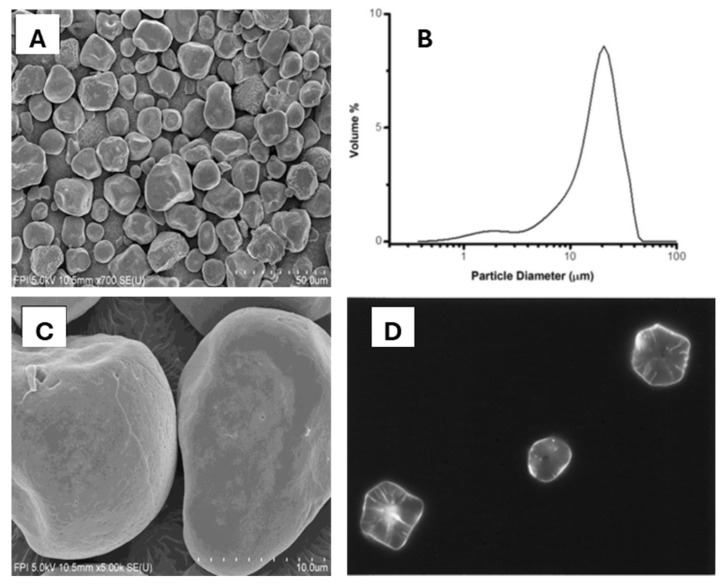
(**A**) Scanning electron microscope images of sorghum starch granules of varying shape and size [39]; (**B**) size distribution of sorghum starch granules as measured by laser diffraction sizing [45]; (**C**) scanning electron microscope images of sorghum starch granules [39]; (**D**) fluorescence microscope photomicrograph of whole sorghum starch granules treated with merbromin methanolic solution and mounted in immersion oil [46]. (Figure 2A). Reprinted from Food and Bioproducts Processing, Volume 91, Belhadi et al., Three small-scale laboratory steeping and wet-milling procedures for isolation of starch from sorghum grains cultivated in Sahara of Algeria, pp. 225–232, 2013, with permission from Elsevier. (Figure 2B). Reprinted from Structure and composition of the sorghum grain. Bean et al. In Sorghum: State of the Art and Future Perspectives, pp. 173–214, Agronomy Monograph 58, Copyright in 2019 by American Society of Agronomy, Crop Science Society of America, and Soil Science Society of America. (Figure 2C). Reprinted from Food and Bioproducts Processing, Volume 91, Belhadi et al., Three small-scale laboratory steeping and wet-milling procedures for isolation of starch from sorghum grains cultivated in Sahara of Algeria, pp. 225–232, 2013, with permission from Elsevier. (Figure 2D). Reprinted from Cereal Chemistry, Volume 74, Huber & BeMiller, Visualization of channels and cavities of corn and sorghum starch granules, pp. 537–541, Copyright 1999–2025 by John Wiley & Sons, Inc.

Sorghum starch granules vary in shape and size. Shapes of sorghum starch granules are polygonal, round, irregular or semi-spherical, and oval or semi-oval [39,40,42,43] (Figure 2A). The sizes of sorghum starch granules range from 4 μm to 35 μm [44] with mainly bimodal or trimodal size distributions [45] (Figure 2B). Yan et al. [43] reported a range of 2 μm to 24 μm and an average of 14.5 μm for sorghum starch particle size when viewed by scanning electron microscopy.

Sorghum starch granules have pores on their surface, leading to channels and then to a central cavity in the starch granules [39,47] (Figure 2C). Figure 2D shows sorghum starch granules treated with a methanolic solution of merbromin and viewed using fluorescence microscopy. The merbromin solution stained the channels and the hilum (starch granule core) through the surface pores on the starch granules. Pores and channels can increase the effective surface area and facilitate the rapid diffusion of amylases to substrates in starch granules [48], thus higher densities of channels lead to higher digestibility of sorghum starch [49].

**Figure 3:**
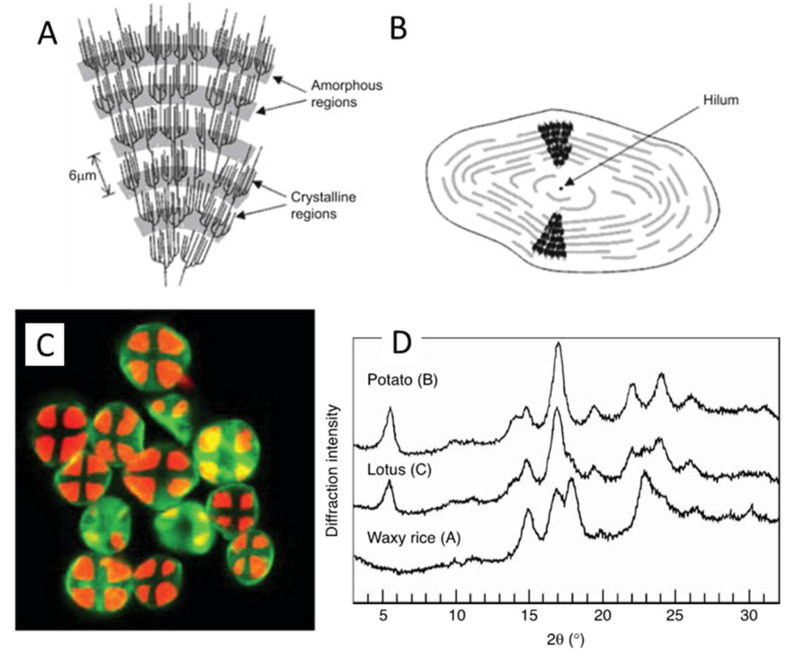
Crystallinity of starch granule. (**A**) Organization of the amorphous and crystalline regions of a starch granule [68]; (**B**) orientation of the amylopectin molecules within a starch granular cross section [68]; (**C**) the Maltese cross of sorghum starch as shown by a Confocal laser scanning micrograph taken with a polarized plate [56]; (**D**) X-ray diffraction pattern of starch with different crystal types [69]. (Figure 3A,B). Used with permission of Royal Society of Chemistry from Polysaccharides in Food: The Chemistry of its Components, 7th edition, Coultate, [68]; permission conveyed through Copyright Clearance Center. (Figure 3C). Reprinted from Pressure-induced gelatinization of starch in excess water, Vallons et al., [56] Critical Reviews in Food Science & Nutrition, published on 4 November 2013, with permission of the publisher (Taylor & Francis Ltd., https://www.tandfonline.com). (Figure 3D). Used with permission of Taylor & Francis Group LLC—Books from Starch: Analytical aspects in Carbohydrates in Foods, 2nd edition, (A. C. Eliasson, ed.) Hizukuri et al., [69], permission conveyed through Copyright Clearance Center.

All starch granules contain both crystalline and amorphous regions and are semi-crystalline. The amorphous part consists of almost all the amylose and the clustered branch points of amylopectin [50,51], and the crystalline part consists primarily of the linear portion of the B1 chains and the A-chains in amylopectin [50]. The crystalline regions of double helical chains and the amorphous branch-point regions in amylopectin are organized into alternating lamellae with a total repeat distance of approximately 9 nm (Figure 3A) [50,52,53,54]. The granules are organized into concentric rings radiating out from the central hilum to the surface of the granule (Figure 3B) [50,55]. Most native starch granules exhibit a characteristic Maltese cross with clear birefringence when viewed with polarized light [42,56] (Figure 3C). This is seen as a radial orientation of the principal axis of the crystallites [51]. The amylopectin molecules inside starch granules have been shown to crystallize into A-type (most cereal starches), B-type (tuber, high amylose cereal starches and retrograded starches) and C-type (legume starches), which represent a combination of A and B-types [57,58,59] (Figure 3D). Sorghum is classified as an A-type starch [60] but this type can change to V-type upon extrusion [61] and similar to B-type upon high pressure processing at 600 MPa [62]. A V-type diffraction pattern could also be observed from amylose–lipids or iodine, alcohols complexes [63]. The main causes of differences between these crystallites are the type of packing of double helices and the number of water molecules in the crystal unit cell [64,65,66,67].

### 2.2. Sorghum Protein

Proteins are sequences of amino acids combined by peptide bonds. Peptide bonds in proteins can be digested (broken down) by proteases. Various criteria including solubility in various solvents, high content of certain types of amino acids, and ease of denaturation are often used to categorize proteins. The major protein classes in sorghum grain are albumins, globulins, prolamins, and glutelins [70,71]. The sorghum protein kafirin is classified as a prolamin due to its solubility in aqueous alcohol and high content of proline and glutamine [72]. Kafirin, a major storage protein, accounts for 68% to 73% of the total protein in sorghum whole grain flours and 77% to 82% in the sorghum endosperm [73]. However, kafirin has poor nutritional properties due to the low content of dietary essential amino acids including lysine, tryptophan and threonine [74,75]. The kafirins include α_1_-, α_2_-, β-, γ-, and δ-kafirin.

The *Sorghum bicolor* genome sequence (~730-megabase) has been published [76]. Genes have been linked to protein digestibility. The kafirin gene family consists of 27 genes (23 for α-kafirin, 2 for γ-kafirin, 1 for β-kafirin, and 1 for δ-kafirin) [17]. On chromosome 5, three natural α-kafirin alleles have been linked to high protein digestibility and one α-kafirin allele has been linked to low protein digestibility in sorghum [77]. Although the genetic and molecular mechanism controlling sorghum protein digestibility is not well understood, it likely includes genes leading to disulfide bond formation and modulation [78]. Techniques to increase sorghum protein digestibility will be discussed in Section 4.2.

The composition, structure (primary, secondary, and tertiary), and size of the various kafirin proteins have been reported. Kafirin contains high amounts of glutamic acid (28.2–30.5%), leucine (17.5–19.2%), alanine (11.8–12.5%), and proline (10.0–10.2%) [79]. Zhu et al. [80] reported that α-kafirin contains 267 amino acid residues, β-kafirin 192 residues, γ-kafirin 186 residues, and δ-kafirin 147 residues, and sequence similarity was less than 42.2% for these proteins. The abundance of predicted type of secondary structure of sorghum flour protein, whole kafirin, and individual kafirins has been summarized in Table 2. Kafirin has a higher total proportion of α-helix and β-sheet structure than zein (70.53% versus 52.05%, respectively) [81]. There are four disulfide bonds in γ-kafirin and in β-kafirin, and five cysteine residues in δ-kafirin without intra-chain disulfide bonds [82]. Protein-modeling procedures were used to generate the 3D tertiary structures of α_1_-, α_2_-, β-, γ-, and δ-kafirin [80,82]. Sizes of individual kafirins have been summarized in Table 3.

Kafirin and zein (the prolamin storage protein of maize) are very similar to each other [84]. Both zein and kafirin can be further classified as α-, β-, γ-, and δ- based on differences in molecular weight, structure, and solubility. The molecular weights of zein sub-fractions are as follows: α-zein (22,000, 18,000, and 16,000), β-zein (14,000), γ-zein (16,000 and 27,000) [85], and δ–zein (10,000) [86], while the molecular weights of kafirins in sorghum are as follows: α-kafirin (26,000–27,000), β-kafirin (18,745), γ-kafirin (20,278), and δ-kafirin (12,961) [87]. A DNA-derived sequence for δ-kafirin protein was shown to have high homology with the δ-zein except for the absence of part of the methionine-rich region [87,88] and has subsequently been identified at the protein level as δ-kafirin [89]. Depending on whether it is floury or vitreous, the sorghum endosperm contains about 66–84% α-kafirin, 7–13% β-kafirin, and 9–21% γ-kafirin [87].

**Figure 4:**
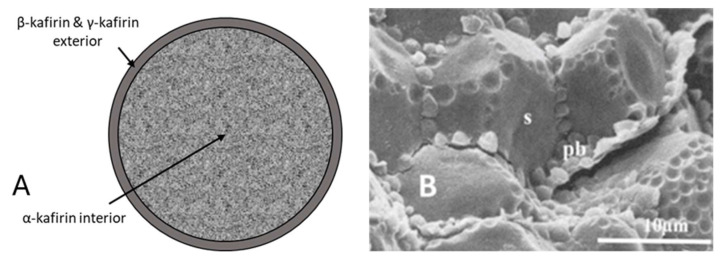
(**A**) Schematic of a sorghum protein body; (**B**) scanning electron micrograph showing sorghum corneous endosperm [93]. “s” designates starch granules and “pb” designates protein bodies. (Figure 4B) reprinted from Morphology, physical, chemical, and functional properties of starches from cereals, legumes, and tubers cultivated in Africa: A review, in Starch/Stärke Emmambux and Taylor, John Wiley and Sons. Copyright © 2013 WILEY-VCH Verlag GmbH & Co. KGaA, Weinheim.

The prolamins in sorghum and maize form protein bodies. The α-prolamin in both cereals is the major storage protein and is in the center of the protein bodies. The β- and γ-prolamins are structural proteins that are usually located at the periphery or within dark-staining inclusions of protein bodies [73,90,91,92]. The schematic, coupled with the scanning electron micrograph in Figure 4A,B, provides a visual representation of the anatomical features of kafirin protein bodies and their orientation relative to the starch. Emmambux and Taylor [93] also demonstrated that the spherical protein bodies are embedded within a glutelin protein matrix and surrounded by starch. Due to a high cysteine content, γ-kafirin tends to form disulfide cross-links that may obstruct protein digestion [94].

Protein and amino acid content of sorghum are presented in Table 4. Mokrane et al. [95] reported that essential amino acids constituted between 40 and 43% of the total amino acids in seven Algerian sorghum cultivars.

The properties of kafirin affect its utilization. Amino acids and proteins in sorghum play a crucial role in the nutritional quality of sorghum for both human foods and animal and aquaculture feed [74,105,106,107]. This review will emphasize methods to improve both protein and starch digestibility for adequate nutrition.

## 3. Endosperm Structure

**Figure 5:**
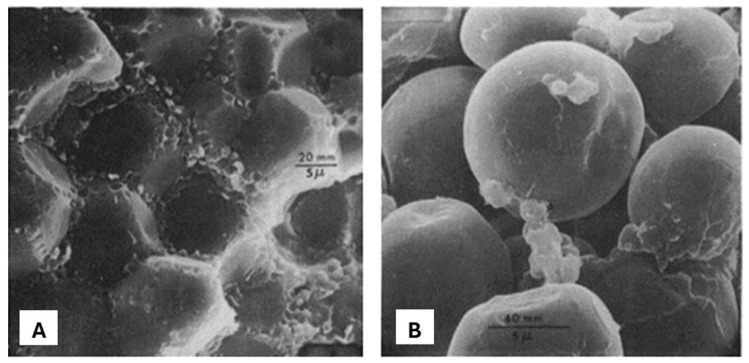
Scanning electron micrograph of (**A**) vitreous and (**B**) opaque endosperms of sorghum grain [6]. Figure 5. Used with permission of John Wiley & Sons—Books from Pericarp and endosperm structure of sorghum grain shown by scanning electron microscopy, Cereal Chemistry, vol. 51, pp. 552–558, Hoseney et al., [6]; permission conveyed through Copyright Clearance Center, Inc.

Cereals such as sorghum and maize have two types of endosperms that are known as the horny (vitreous, corneous, or hard) and floury (opaque or soft) endosperms as shown in Figure 1. These classifications are based upon the arrangement of the endosperm components. The microstructures of vitreous and opaque endosperm regions are different as shown in Figure 5.

### 3.1. Vitreous Endosperm

The vitreous endosperm contains polygonal-shaped starch granules. Matrix proteins can be defined as proteins originating from the vitreous endosperm that form a continuous network around starch granules and kafirin protein bodies [108]. The starch is surrounded by protein bodies and protein matrix with a continuous interface between starch and protein. No visible air spaces exist among the components of the vitreous endosperm (Figure 5A). The presence of the protein bodies causes indentations in the starch granules [35]. The density of this packing arrangement results in a translucent ‘glassy’ appearance of the vitreous endosperm regions [6].

### 3.2. Opaque Endosperm

The opaque endosperm, on the other hand, consists of loosely packed cells with spherical starch granules. The protein distribution in the opaque endosperm is different than the distribution in the vitreous endosperm. The protein matrix in opaque endosperm is discontinuous, with protein bodies embedded in the matrix. Air spaces exist between the cell components and cause the discontinuity of the protein matrix (Figure 5B) [6]. The presence of air spaces within the endosperm causes a diffraction of incident light, resulting in a distinct opaque appearance.

## 4. Sorghum Starch and Protein Digestibility

### 4.1. Sorghum Starch Digestibility

This next section will discuss sorghum starch digestibility throughout the gastrointestinal tract, on a granular level, and at the enzymatic level. The effect of starch digestion on blood glucose levels, ways to measure digestibility, and classification of starch based on digestibility are considered next.

In vivo starch digestion occurs in several steps throughout the gastrointestinal tract including in the mouth, stomach, small intestine, and large intestine [109,110]. Mastication (or reduction in food particle size by chewing to form the food bolus) and salivation (for initial α-amylase hydrolysis of starch into shorter oligomers within the saliva) occur in the mouth. In the stomach, gastric emptying, gastric motility, pepsin hydrolysis, and lipase hydrolysis affect starch digestion. Within the small intestine, starch is mainly digested into smaller oligomers by pancreatic α-amylase within the duodenum. The resulting mixture of oligosaccharides (maltose, maltotriose, and oligoglucans) passes through the mucous layer and brush border membrane to be further degraded to glucose by α-glucosidases. Glucose absorption occurs in the jejunum and ileum through the epithelial cells by indirect active co-transport and facilitated diffusion mechanisms. Small intestinal gut motility and transit time also affect digestion and absorption of starch. Gastric emptying is regulated by hormones such as GLP-1, cholecystokinin, and PYY. Within the large intestine, resistant starch could act as a prebiotic and be fermented under anaerobic conditions by the gut microbiota to produce short-chain fatty acids such as acetate, propionate, and butyrate. Butyrate is used by the colonic epithelium as an energy source, and propionate and acetate are transported to the liver and used as substrates for lipogenesis and gluconeogenesis. The short chain fatty acids can also bind to G-protein-coupled receptors to activate various hormones [110].

Starch digestion on a granular level has been described by a series of steps. First, enzymes randomly diffuse onto starch granular surfaces. Second, enzymes move by capillary action into the starch granules. Third, enzymes begin to hydrolyze the starch at their contact points. Fourth, the hydrolysis continues radially and forms more pores and channels towards the granular core. Finally, the hydrolysis spreads from the granular core [111]. Surface pores on sorghum starch granules were observed by scanning electron microscopy before fermentation but enlarged to become large pin holes that allow enzymes to diffuse and hydrolyze starch granules after 24 h of fermentation [107,112].

The three-dimensional structures of human salivary α-amylase and human pancreatic α-amylase have been determined. Both enzymes contain 496 amino acid residues, and only 14 of these residues are different between these two amylases [109,113]. Also, there are three structural domains and a binding site for chloride and calcium ions. The A domain forms a barrel structure and contains an Asp 197, Glu 233, and Asp 300 active site (catalytic) residues [109,113]. The carboxyl groups on aspartate and glutamate residues participate in acid/base catalysis and nucleophilic catalysis [114]. More details about amylases and related enzymes can be found in [115].

Many factors affect starch digestion. Mahasukhonthachat et al. [116] reported that granule properties including size, degree of crystallinity, degree of polymerization, non-starch components, interactions with starch, amylose to amylopectin ratio, and type and extent of processing affect starch digestion. Additional factors affecting starch digestion include endosperm structure, granular architecture and protein body distribution, and structure and chain length distribution of amylopectin.

The endosperm structure of sorghum flour affects its in vitro digestibility. Sorghum starch digestion by porcine pancreatic α-amylase is significantly higher in opaque endosperm (94%) than in vitreous endosperm (87%) [117]. Although sorghum and maize endosperm structures are similar, digestibility of maize flour does not appear to be affected by whether it is floury or vitreous in origin [117]. γ- and β-Kafirin, rich in cysteine, are found in greater abundance in the vitreous endosperm than the opaque endosperm [94,117]. This makes the formation of polymeric kafirin more prevalent in the vitreous endosperms due to enhanced intermolecular disulfide bonding during processing. Cross-linking among γ- and β-kafirin and matrix protein impedes starch gelatinization and digestibility [117]. Interestingly, the kinetic constants of enzymatic hydrolysis of both opaque and vitreous endosperms of sorghum are similar, denoting that extrinsic factors are the primary reasons for low digestibility of sorghum starch [117]. Another report also indicated that sorghum protein is the key component affecting starch digestibility [118].

The proportion of amylose to amylopectin in sorghum grain is another factor influencing starch digestion [7], gelatinization, and granular swelling. The wx phenotype has been recognized in sorghum since 1933 [119], and the wx genes have been cloned in 1996 [120]. Increased incremental number of recessive waxy endosperm genes leads to an increase in vitro sorghum starch hydrolysis [121]. The level of waxiness positively correlates with the in vitro digestibility of raw sorghum starch [27]. Waxy sorghum also exhibits more extensive gelatinization during extrusion than high amylose sorghum [61]. Waxy starches generally have much greater granular swelling than normal sorghum during cooking.

Amylose could play a role in restricting granular swelling to cause poor digestibility of amylose-containing sorghum starches [122] by forming complexes. Amylose can impede gelatinization by forming amylose–lipid complexes which increase the rigidity of granules [27]. Gomez et al. [61] reported that extrusion changes the X-ray diffraction pattern of sorghum from an A-type to V-type, suggesting that amylose is forming complexes with organic molecules, such as lipids. This complex formation makes it harder for starch-hydrolyzing enzymes to reach the starch inside the complex, thereby reducing digestibility. The single helix of long chain amylose with high hydrophobicity in the center makes amylose prone to clathration with organic compounds to form helical inclusion. Amylose–lipid complexation improves the stability of amylose, so the molecule is in the state of minimum energy. Hydrophobic interactions are predominantly involved in forming helical inclusion [123]. Lipids form inclusions with amylose, with the hydrocarbon portion of the lipid located within the helical cavity of amylose [123]. The amylose–lipid complexes also decrease water-solubility besides interfering with the hydrolytic activity of starch-degrading enzymes. Heating enhances the formation of amylose–lipid complex [124]. Hence, the presence of amylose in sorghum influences the digestibility of starch.

However, the ratio of amylose to amylopectin does not directly influence the hydrolysis of starch in the granular state (raw starch), but the granular architecture and protein bodies play important roles in the differences in starch digestibilities. In granular starch, the difference in hydrolysis of wx-type starch compared to non-wx starch is due to the granular architecture, including the surface holes, channels, and crystalline organization, rather than the molecular structure [125]. In the case of sorghum, the kernel structure is responsible for the difference in digestibility between amylose containing and non-amylose containing grains. The structures of vitreous endosperm in wx and non-wx sorghum are different. The protein bodies in wx sorghum are more evenly distributed compared to normal sorghum. In varieties of sorghum with higher amylose content, the protein bodies are more concentrated in the vitreous endosperm than in the opaque endosperm [61]. The protein bodies in normal sorghum are more numerous and more tightly associated with starch granules compared to wx sorghum [126]. A high concentration of protein in this endosperm reduces the accessibility of starch to enzymes due to the formation of starch–protein matrices [127]. This could also explain the low digestibility of amylose-containing sorghum grains.

The structure and chain length distribution of amylopectin influence the porosity and digestibility of starch granules. The structure of amylopectin influences the shape and the architecture of the starch granule. Variation in amylopectin structure can result in starch granules with different physical properties and morphologies [128]. Sorghum has significantly smaller proportions (12.8–14.0%) of short chains with a degree of polymerization (DP) of 12 compared to maize (15.0%) [129]. These are external chains that include both A- and B1-chains of amylopectin. Sorghum amylopectin was also found to have an equal or larger proportion of chains with DP 13–24 (41.3–43.3%) than maize amylopectin (41.3%) [129]. Amylopectin with greater proportion of short chains results in more porous starch granules, which makes the granule more susceptible to enzyme [130]. Low proportion of short chains in sorghum may make the starch granules less porous than maize granules, contributing to poor starch digestibility in the granular form.

The kinetics, effect of particle size, and extent of sorghum starch digestion have been studied. Mahasukhonthachat et al. [111] has shown that in vitro starch digestion in ground sorghum closely follows first-order kinetics. Sorghum starch digestibility generally increased with decreasing particle size, presumably due to increased relative surface area [111]. The square of the particle size had a significant linear relationship with the reciprocal of the digestion rate constant, consistent with a diffusion-controlled mechanism for digestion [111]. Al-Rabadi et al. [131] found that starch digestibility was incomplete after 24 h of incubation, possibly due to the presence of a protein matrix or the binding of amylase inhibitors such as polyphenols and phytate.

The rate of starch hydrolysis depends on the form of the starch granules. The highly ordered molecular structure of native starch is only slowly digested by enzymes [132]. In the presence of water and sufficient heat, the intermolecular bonds of starch molecules within starch granules breakdown, causing starch granule swelling, melting of double helical structures, and leaching of amylose in a process called gelatinization. Gelatinization leads to more rapid breakdown of starch by hydrolyzing enzymes such as α-amylase. However, sufficient cooling of gelatinized starch will lead to rearrangement into a more crystalline structure in a process called retrogradation to restrict the availability of amylase to hydrolyze starch [132].

Ways to increase starch digestion include further improving enzyme access to starch, increasing rate of digestion after the enzyme and starch are in contact, and eliminating potential amylase inhibitors. Possible ways to accomplish these tasks include using protein or cell wall degrading enzymes, efficient starch gelatinization without subsequent retrogradation, and eliminating tannins [111].

Blood glucose levels rise after starch digestion and absorption and are affected by GI (glycemic index) and GL (glycemic load) of the consumed food. The corresponding increase in blood glucose levels after food or glucose consumption can be monitored over two hours, and the percentage of the area under this curve due to this food consumption relative to the area under this curve due to the equivalent amount of glucose consumption is referred to as the glycemic index (GI) [133]. GI of foods can be calculated by the area under the digestogram as described by [134]. GL is calculated by multiplying the grams of carbohydrate available in the food by the GI and then dividing this product by 100, so GL considers both the quality and quantity of carbohydrates consumed [135]. The GI of foods can range on a scale of 1 to 100 and can be categorized as high (GI > 70), medium (GI between 56 and 69), or low (GI < 55) [136]. GI is typically low and variable in foods containing RS as RS does not breakdown in the small intestine [137]. Instead of measuring GI in vivo, GI can be estimated by an in vitro procedure [138].

There are advantages and disadvantages for measuring GI in vivo or estimating GI in vitro. An advantage for measuring GI in vivo is that it is intended to represent the digestive physiology in humans from chewing food, starch breakdown in the mouth and small intestines, and glucose absorption from the gastrointestinal tract into the bloodstream [135]. However, it is difficult to adequately model this complex digestion process. Other advantages for measuring GI are that there is an International Standards Organization standardized test method for this test, and there are tables of GI values available in the literature [139]. Conversely, there are various procedures for assessing in vitro starch digestibility, making it difficult to compare results from different procedures and different laboratories. If possible, in vitro methods should be validated against in vivo GI values for the same food. Disadvantages for measuring GI in vivo include biological variability between test participants including rate of gastric emptying, limitations on number of test participants and precision of results, inability to test non-foods, and difficulty in controlling other variables. Corresponding advantages for in vitro starch digestibility procedures are better control over experimental conditions, ability to use larger sample sizes due to lower labor requirements, and the ability to test non-foods [135]. In order to compare digestibility results between different studies, it would be beneficial to standardize in vitro and in vivo digestibility methodologies.

Numerous studies have reported the GI or estimated GI values and factors affecting these values of sorghum-based products. Mabureza et al. [100] reported estimated GI values ranging from 68.76 to 74.56 for four cultivars of sorghum grain from Mozambique. Moraes et al. [41] reported estimated glycemic index values of 84.5 for decorticated sorghum flour (prepared using a rice polisher), 77.2 for whole sorghum flour, and 60.3 for sorghum bran, and they found that resistant starch was not correlated with estimated glycemic index. Pruett et al. [140] made muffins using whole grain sorghum flour ground to fine (82.2 μm), intermediate (166.9 μm), or coarse (303.3 μm) mean particle sizes and measured mean in vivo GI of 56, 32, and 50, respectively, from participating volunteers. Prasad et al. [141] prepared various sorghum-based and wheat-based products (multigrain *roti*, coarse semolina *upma*, fine semolina *upma*, flakes *poha*, pasta, and biscuits) and measured the resulting GI. They reported GI values ranging from 45 to 68 for the sorghum-based products, and all these sorghum-based products except for multigrain *roti* and biscuits had lower GI values than their wheat-based counterparts. Jiang et al. [142] prepared a sorghum-based snack enriched with both soy protein isolate and soy flour, a sorghum-based snack enriched only with soy flour, and a control sorghum-based snack and found that the estimated GI decreased in the order of the control sorghum-based snack (79.9), the sorghum-based snack enriched only with soy flour (70.6), and the sorghum-based snack enriched with both soy protein isolate and soy flour (59.8). Mahasukhonthachat et al. [116] found that the AUC_exp_ (the area under the digestogram for the modified first-order kinetic model) and the AUC_Peleg_ (the area under the digestogram for the Peleg model) of sorghum extrudates was approximately three times higher than the AUC_exp_ and AUC_Peleg_ of non-extrudates.

There are different procedures for measuring starch digestibility besides GI and estimated GI. Starch digestion has been measured in vivo in ileostomic patients [143] and estimated by the release of glucose measured in the lab by various in vitro procedures [144,145,146,147]. Englyst et al. [144] originally classified starch into rapidly digestible starch (RDS), slowly digestible starch (SDS), and resistant starch (RS) based upon the amount and timing of glucose release when the starch is hydrolyzed in vitro with a series of enzymes. These enzymes have included pepsin, pancreatin, heat stable α-amylase, pullulanase, amyloglucosidase, and invertase to mimic starch digestion in humans. In the most recent protocol, RDS is determined by measuring the amount of glucose released from starch hydrolyzed by addition of pancreatin amylase and amyloglucosidase at 37 °C within 20 min. The RDS correlates with the rapid increase in the blood glucose level after ingestion of carbohydrate containing foods. The SDS is determined the same way as determining RDS but at both 20 and 120 min and then subtracting the amount of glucose released from starch up to 20 min from the amount of glucose released from starch up to 120 min. In the original definition, RS is starch that is unhydrolyzed after 120 min, and RS is meant to represent starch that is not digested in the small intestine but passed into the large intestine for fiber utilization via fermentation by colonic microflora [148]. The RDS, SDS, and RS parameters are often expressed as percentage of the total starch in the sample. In an early procedure for measuring RS, a 16 h incubation time with pancreatic α-amylase and amyloglucosidase was used to solubilize and hydrolyze non-RS to glucose [145]. Afterwards, the sample incubation time with pancreatic α-amylase/amyloglucosidase mixture was reduced from 16 h to 4 h to more closely represent the transit time of food through the human small intestine [146]. The term “total digestible starch” (TDS) was then introduced to quantitate the amount of glucose released from the starch that is hydrolyzed within 4 h of incubation with a pancreatic α-amylase/amyloglucosidase mixture, and the definition of RS was modified to the amount of unhydrolyzed starch remaining after 4 h of incubation, instead of 120 min (2 h), with a pancreatic α-amylase/amyloglucosidase mixture.

Brodkorb et al. [149] reported a standardized protocol called INFOGEST 2.0 based on a static digestion method for assessing endpoints of food digestion by analyzing digestion products. Constant ratios of meal to digestive fluids and a constant pH at each step are used for sequential oral, gastric, and intestinal digestion using available physiological data for electrolytes, enzymes, bile, dilution, pH, and digestion time [149].

Various values for RDS, SDS, TDS, and RS of sorghum products have been reported in the literature and presented in Table 5, and these digestibility values can affect ethanol yields. Sorghum is recognized as a slowly digestible cereal grain due to poor starch digestibility [150] caused by the structure of the endosperm, nature of sorghum protein, presence of amylose and enzyme inhibitors. Also, the RS content of raw sorghum grain is normally higher than the RS content of other cereal grains [151]. Elkonin et al. [32] found that the sorghum line with the highest total starch content (82.17%) in their study had the lowest TDS value (45.74%) but a sorghum line with nearly the lowest total starch content (64.58%) had the highest TDS value (69.25%). Yang et al. [152] measured values of 89.89 to 93.11% for RDS, 3.61 to 7.37% for SDS, and 2.56 to 3.39% for RS for six varieties of red sorghum with varying amylose levels. Their low-amylose varieties had the lowest RDS and RS contents but the highest SDS contents. The high starch digestibility of their low-amylose varieties led them to believe that these varieties would lead to higher ethanol yields for baijiu production (a colorless Chinese liquor typically made from fermented sorghum and containing between 35 and 60% alcohol by volume). Likewise, Weiss et al. [153] reported that higher ethanol fermentation efficiency can be obtained when fermenting waxy sorghum instead of normal sorghum.

There are five types of resistant starch (RS) [26,157,158]. RS1 is physically inaccessible starch mainly inside cell walls that is unfractionated or unrefined but may be fully digested when properly milled. Sources include whole or partially milled grains and seeds, legumes, pasta, raw fruits, and vegetables. RS2 is native starch granules (inadequately gelatinized or uncooked) with a highly crystalline structure mainly found in raw potato and green (unripe) bananas but also present in high amylose corn starch, cereals, raw legumes, raw fruits, and vegetables. RS3 is retrograded starch such as cooked and cooled potato, retrograded high amylose corn starch, bread crust, corn flakes, and foods exposed to repeated moist heat treatments. Retrogradation occurs when starch is cooked above its gelatinization temperature in water and subsequently cooled. RS4 is starch that has been chemically or physically modified by adding bulky functional groups to restrict access of α-amylase into starch granules, thereby increasing its resistance. This type of starch does not occur naturally but can be used to produce high-fiber drinks, breads, and cakes. RS5 is a starch with a high amylose content that has formed amylose–lipid complexes (or other type of complexes such as starch–glycerol, starch–protein, starch–peptides, starch–amino acid, starch–lipid–protein, and starch–polyphenol complexes) by hydrothermal treatments. These complexes within starch granules may restrict granular swelling during cooking to increase enzymatic resistance to hydrolysis [26,158].

### 4.2. Sorghum Protein Digestibility

This section will discuss procedures for measuring protein digestibility, values of protein digestibility, and factors affecting protein digestibility. Modifications to sorghum protein digestibility will then be covered.

Procedures have been published for determining protein digestibility. Diatta-Holgate et al. [159] described a protocol as grinding the seed with water into flour, cooking the sorghum at 95 °C for 20 min into porridge, digesting the porridge with pepsin at 37 °C for 2 h, extracting the protein extract, and assaying it spectrophotometrically at 562 nm. This procedure is a modification of an earlier procedure [160] by adding a cooking step, increasing the pepsin incubation time from one to two hours, and taking absorbance readings at 20 min instead of various times up to 60 min. An earlier assay by Aboubacar et al. [161] was based on measuring the amount of undigested protein remaining after one hour of pepsin digestion by performing SDS-PAGE and image analysis of the gels. Two other procedures that can be used for measuring protein digestibility in sorghum include use of a modified pepsin method and a sulfuric acid digestion step by Mertz et al. [162] and an enzymatic pH-stat procedure using trypsin, chymotrypsin, and peptidase by Pederson and Eggum [163].

Many values for in vitro sorghum protein, protein concentrate, and protein hydrolysate digestibility are presented in Table 6. Percentages of in vitro protein digestibilities of fine red sorghum flour (86.02%) and fine white sorghum flour (86.38%) were slightly higher than percentages of in vitro protein digestibilities of red sorghum gluten meal (84.85%) and white sorghum gluten meal (85.30%) [164]. The lower digestibilities of the meals may be explained by exposure of the internal protein structure to anti-nutrients such as tannins during the wet milling process and the high-temperature extraction process [164]. Wulandari et al. [165] used a combination of chymotrypsin, pancreatin, and trypsin enzymes for determining in vitro protein digestibilities and reported values of 13.18% for sorghum grains, 30.80% for sorghum protein concentrates, and 47.14% for sorghum protein hydrolysates produced with bromelain enzyme.

Many factors affect protein digestibilities of kafirin and extracted kafirin. The in vitro protein digestibilities of flour from 15 genotypes of sorghum in the study of Weerasooriya et al. [97] averaged 48.7% (with a range of 27.7% to 75.1%) and was negatively correlated with their total protein, total kafirin, and γ-kafirin content. These authors explained the inverse relationship between γ-kafirin content and in vitro protein digestibility by the high cysteine content in γ-kafirin, leading to disulfide bonds. The high lysine genotype, Wetet Be-gunchie, in their study also had a low γ-kafirin content but a high in vitro protein digestibility [97]. In addition to kafirins reducing starch digestion, Elkonin et al. [32] reported that kafirin digestion by pepsin can be reduced by previous starch digestion by amylolytic enzymes. Espinosa-Ramírez and Serna-Saldívar [169] measured in vitro protein digestibilities of kafirin-rich protein extracts from various types of whole and decorticated sorghum genotypes and found that extracted kafirins had in vitro protein digestibilities ranging from 86.54% for whole white-regular sorghum to 90.82% for decorticated white-waxy sorghum. The decorticated sorghums tended to have slightly higher protein digestibilities than whole sorghums in their study.

Altering sorghum protein affects its digestibility. Benmoussa et al. [170] developed a high-lysine mutant P721Q (*hdhl*) sorghum with high protein digestibility. Increased α-kafirin but decreased γ-kafirin were produced, resulting in different packing of these subunits and forming a multi-folded protein body [170]. The digestibility of γ-kafirin has been improved by forming a mutant by introducing a genetic construct that induces RNA silencing of the γ-kafirin gene [171]. These authors were able to improve the protein digestibility from 52 to 59% in the original cultivar up to 81% in one line. Li et al. [172] used CRISPR/Cas9 gene editing to target the K2G gene in sorghum encoding γ-kafirin to decrease the γ-kafirin content by 12.75 to 19.22% and increase the protein digestibility by 26.91 to 74.31% in the mutants compared to the wild type.

## 5. Health Benefits Provided by Sorghum and RS/SDS Starch Consumption

While the previous sections have established the fundamental aspects of sorghum composition and digestibility mechanisms, the following section explores how these properties translate into specific health benefits. Understanding the relationship between sorghum’s digestibility characteristics and its physiological effects is essential for optimizing its use in both traditional diets and functional food applications.

### 5.1. General Health Benefits

Sorghum consumption provides many potential health benefits and sorghum contains bioactive compounds. These potential health benefits found by various in vitro, cell culture, lab animal, and human studies include antioxidant activity, anti-inflammatory activity, anti-microbial activity, anti-diarrheal activity, cancer prevention, maintaining satiety, inhibiting α-glucosidase activity, prevention of diabetes and obesity, and prevention of cardiovascular disease including lowering of plasma cholesterol and triacylglycerol levels [10,173,174,175]. Although antioxidant activity is typically measured by colorimetric in vitro assays, Wu et al. [176] applied a cell culture to quantify antioxidant activity in a more physiologically relevant condition and found increased cellular antioxidant activity of Chinese steamed bread incorporating sorghum. Shim et al. [177] induced ear edema in mice with 12-*O*-tetradecanoylphorbol-13-acetate (to stimulate inflammation) and found less ear inflammation in mice fed golden gelatinous sorghum extract than in the control mice. Sorghum has been shown to reduce blood sugar levels [178] by mechanisms including improving insulin sensitivity [179] and inhibiting starch digestion and hepatic glucose production [173]. Consumption of an extruded whole grain sorghum beverage, with or without added *Lacticaseibacillus* (formerly *Lactobacillus*) *paracasei*, by overweight and obese adult subjects led to their improved body composition, Castelli index I scores (atherogenic index, equal to total cholesterol divided by high-density lipoprotein cholesterol), and intestinal health compared to subjects consuming a control waxy maize starch beverage [180]. Sorghum is rich in phytonutrients, especially 3-deoxyanthocyanins and tannins in colored sorghum [175]. Bioactive compounds found in sorghum include phytosterols, carotenoids, and phenolic compounds [181]. Also, fatty acid esters of hydroxy fatty acids are bioactive lipids and have been identified in sorghum [182]. Based on the antioxidant activity of sorghum and the bioactive compounds that it contains, products incorporating sorghum grains can be considered as functional foods [181].

### 5.2. Benefits of Low GI and High SDS/RS

The GI and GL of an individual’s diet can affect their health. Schwingshackl et al. [183] performed a meta-analysis on the relationship of GI/GL to various parameters related to being overweight and obesity in children and adolescents and found that low GI diets led to greater decreases in serum triglycerides and HOMA (homeostatic model assessment of insulin sensitivity) index than high GI diets. Livesey et al. [184] concluded that a diet of high GI and GL probably causes type 2 diabetes. Jenkins et al. [185] evaluated the diets of 137,851 participants from five continents and their subsequent cardiovascular diseases and deaths and concluded that diets that were characterized by a high GI led to increased incidences of cardiovascular disease and death. Turati et al. [186] concluded that a high GI diet led to moderately unfavorable effects on colorectal cancer, and possibly also on bladder and kidney cancers, versus a low GI diet and that an increased GL diet may have been modestly associated with increased incidence of endometrial cancer.

There are various health benefits provided by SDS and RS. SDS provides health benefits by stabilizing blood glucose levels and improving the GI, leading to a decreased risk of diabetes and cardiovascular disease [187]. RS, including RS3, consumption also provides many health benefits as it enters the large intestine to potentially function as a prebiotic by fermentation to short chain fatty acids (acetate, propionate, and butyrate) by the gut microbiota [110,188]. Li et al. [189] showed that a diet containing RS supplementation led to weight loss with changes in gut microbiota composition, especially for *Bifidobacterium adolescentis*. RS assists in preventing colon cancer, has hypocholesterolemic and hypoglycemic effects, reduces gallstone formation, promotes ileal absorption of various minerals, suppresses appetite, and inhibits fat accumulation [26,158,190].

### 5.3. Benefits for Celiac Patients

Sorghum can be consumed by celiac patients due to the lack of gluten. Celiac disease is an autoimmune disease that causes gastrointestinal problems such as chronic diarrhea and malabsorption. This disorder is characterized by an inappropriate immune response in susceptible people leading to complex inflammation upon the ingestion of gluten. People with celiac disease cannot consume certain cereal grains such as wheat, rye, and barley [191]. Although celiac patients can safely consume sorghum, its digestibility is reduced upon cooking, especially wet cooking [192]. This low digestibility of sorghum is unfortunate for celiac patients because of the limitations of the foods that they can safely consume. Therefore, it would be highly desirable to increase the digestibility of sorghum proteins and starch.

## 6. Anti-Nutritional Factors Affecting Digestibility

Anti-nutritional factors can adversely affect the ability to digest and absorb essential nutrients. These factors include oxalate, phytate, tannins, and trypsin inhibitors, and the concentration of each of these anti-nutrients in sorghum flour decreased upon fermentation for 48 h [104]. Phenolic compounds such as tannins can inhibit digestive enzymes including α-amylase, α-glucosidase, and trypsin. Elkhalil et al. [193] was able to reduce the phytic acid content in sorghum flour and progressively increase in vitro protein digestibility by a malt pretreatment process. Kafirin inhibits starch digestion, and RS content increased from 5.09 to 21.04% after cooking upon addition of 7.5% kafirin to corn starch [194].

### 6.1. Phenolics

Phenolics can affect in vitro protein digestibility. Sorghum flour and cooked sorghum products originating from sorghum genotypes containing tannins usually had lower in vitro protein digestibility than those products originating from genotypes not containing tannins [97]. Unlike addition of extracts containing phenolics from non-tannin sorghum bran, addition of extracts containing phenolics from tannin sorghum bran lowered in vitro protein digestibility [195]. Tannins in sorghum can interact with digestive enzymes and protein, leading to decreased nutrient digestibility [196]. Tannin-protein complexes may be broken down by the depolymerization of sorghum procyanidin C1 [197].

Phenolics including tannins can also adversely affect in vitro starch digestibility. Both tannin extracts containing phenolics and non-tannin extracts containing phenolics were separately added to white sorghum meal and were found to lower estimated GI and RDS but to raise SDS and RS [195]. Although aggregate formation in sorghum meals without the addition of phenolic extracts was observed after iodine staining and viewing with a bright light microscope in their study, larger aggregates were observed in sorghum meals containing non-tannin and tannin phenolic extracts, suggesting that additional interactions were promoted by the phenolic extracts. The digestion process led to more intensely stained visible aggregates in samples containing phenolic extracts. The accessibility of the digestive enzyme in samples after phenolic extract addition and microwave heat moisture treatment was limited by the compact molecular structure. Baah et al. [195] considered these aggregates as a possible type 5 resistant starch, which is a V type complex, and forms based on starch–protein, starch–polyphenol, and starch–other polysaccharide interactions. Condensed tannins in sorghum interact with amylose, possibly by hydrophobic interactions and hydrogen bonding, and to a lesser extent amylopectin, to increase the RS content of normal and high amylose starches [198].

### 6.2. Enzyme Inhibition

Sorghum phenolic extracts have been shown to inhibit α-amylase. The α-amylase inhibitory effects were only observed for human pancreatic and salivary α-amylases, and the inhibition mode was identified as non-competitive [199]. The inhibitors bind to an alternative site of the enzymes causing conformational changes in the enzyme structure. Proteolytic enzymes such as pepsin present in the human stomach can inactivate the α-amylase inhibitors before the food is digested by human pancreatic α-amylase and mucosal α-glucosidases in the small intestine.

The α-amylase inhibitors have no effect on porcine pancreatic α-amylase and *Bacillus subtilis* α-amylase [199]. Porcine pancreatic α-amylase has been used in in vitro analysis to determine starch digestibility [27,34,35,117,118,144]. Therefore, the low in vitro starch digestibility of sorghum flour is not due to α-amylase inhibitors.

Proteinaceous inhibitors of α-amylase that are present in sorghum are heat-labile [199]. Therefore, thermal processing reduces the activity of α-amylase inhibitors in sorghum. The degree of inactivation varies with thermal processing methods [200].

Trypsin inhibitors are another class of inhibitors found in sorghum [201,202]. Trypsin plays an important role in protein digestion in humans, but the presence of trypsin inhibitors may contribute to the decreased protein digestibility of sorghum flour. Trypsin inhibitory activity between three genotypes tested in raw flour, dry cooked sorghum grain, fermented flatbread, porridge, and unleavened flatbread was not significantly different except for two genotypes used for making porridge [97]. An average trypsin inhibitor activity of 6.27 mg of trypsin inhibited per g of flour sample was found [97]. Like α-amylase inhibitors, trypsin inhibitors are also heat-labile [203]. Boiling sorghum in water for 20 min completely inactivates trypsin inhibitors, but oven heating was not very effective in inactivating these inhibitors [203].

Sorghum consumption may potentially decrease post-prandial hyperglycemia. The activities of α-glucosidase and α-amylase are inhibited by extracts from sorghum [204,205]. This inhibition of these carbohydrate hydrolyzing enzymes may be a therapeutic approach for treating diabetes mellitus.

## 7. Cooking Sorghum

### 7.1. Changes in Digestibility upon Heating/Cooking

Cooking affects cereal digestibility. Typically, cooked cereal grains have higher starch digestibilities than uncooked grains. This higher digestibility can be explained by cooking grains with heat and water causing starch gelatinization that makes the starch more accessible to digestive enzymes. Protein denaturation also occurs during cooking and usually increases the susceptibility of enzymatic hydrolysis of proteins.

However, cooking sorghum typically reduces the digestibility of their proteins [74,75]. The in vitro protein digestibility decreased by 33% and 39% by cooking two Dutch sorghum grain varieties [98]. Likewise, Xu et al. [206] found a decrease in protein digestibility after cooking compared to the native sorghum flours. Also, dry cooking of sorghum grain and cooking of fermented flatbread, porridge, and unleavened flatbread made from sorghum flour decreased in vitro protein digestibility, typically to a greater extent than for corresponding products made from maize [97]. In vitro protein digestibility determinations using pepsin at pH 2.0 and 37 °C revealed reduction in protein digestibility of sorghum flour from 80.7% to 64.8% after cooking [167]. Similar observations were reported where sorghum protein digestibility was reduced from 88 to 93% before cooking to 45–56% after cooking [103]. Cooking decreases digestibility of sorghum proteins likely due to disulfide cross linkage formation between β-kafirins and γ-kafirins on surfaces of protein bodies [207]. However, increased protein digestibility from cooking sorghum has also been reported. Cabrera-Ramírez et al. [197] used in vitro gastrointestinal digestion studies to simulate human physiological conditions in the oral, gastric, and the small intestine stages and found that nixtamalization (alkaline cooking) increased bioaccessibility of protein from red and white sorghum during the intestinal phase but had little effect during the oral and gastric stages.

Variable effects of cooking on sorghum starch digestibility have been reported. Many studies have reported that wet-cooked sorghum has lower starch digestibility than uncooked sorghum [167,208]. Vu et al. [209] reported that the RS content increased from 5.6% for untreated sorghum flour to 22.1% for sorghum flour containing added water giving a moisture content of 20% and subjected to heating at 100 °C for 4 h. However, Xu et al. [206] found an increase in starch digestibility after cooking compared to the native sorghum flours. Likewise, Batariuc et al. [210] found that RDS, TDS, and RS generally increased after applying dry heat treatments between 121 and 140 °C to three size classes (less than 200 µm, between 200 and 250 µm, and greater than 300 µm) of sorghum flour. Peterson et al. [211] heated sorghum flour dispersed in phosphate buffer solutions adjusted to pH values of 3, 4, 5, 7, and 8 at 100 °C for 0 (control), 10, 30, 60, or 120 min and found increased digestible starch but decreased resistant starch at all cooking times and pH adjustments, while preserving phenolic content and bioactivity. These different effects of cooking on sorghum starch digestibility may be explained by differences in the form of sorghum (untreated flour versus flours dispersed in pH-adjusted phosphate buffers) and in the type of cooking (dry heating versus wet cooking).

### 7.2. Gelatinization and Cross-Linking

Cooking sorghum flour with water and heat induces gelatinization of starch granules. During gelatinization, hydrogen bonds in the amorphous region of granules are disrupted. This allows water to associate with free hydroxyl groups and causes swelling of the granule. Gelatinization causes loss of the molecular order, destruction of crystalline structures, loss of birefringence, irreversible granular swelling, and starch solubilization. Loss of birefringence is used as a major criterion for starch gelatinization, indicating disruption of the molecular order.

Upon heating, the protein bodies surrounding starch in the endosperm form crosslinks between prolamin molecules via disulfide bonds. These newly formed crosslinks prevent the access of enzymes to starch. Prolamins may also be involved in forming crosslinks with starch molecules making the starch less accessible to starch hydrolyzing enzymes [208]. Conformational changes in secondary structure of protein (increased antiparallel intermolecular β-sheet character and possibly decreased α-helical conformation) in wet cooked ordinary sorghum and wet cooked highly digestible mutant sorghum were shown to be similar when analyzed with FTIR (Fourier Transform Infrared Spectroscopy) and solid state ^13^C NMR (Nuclear Magnetic Resonance spectroscopy) [212]. Therefore, these authors concluded that changes in the secondary structure are not as important as the invaginated protein body structure to sorghum protein digestibility.

### 7.3. Cooking Sorghum Versus Maize

**Figure 6:**
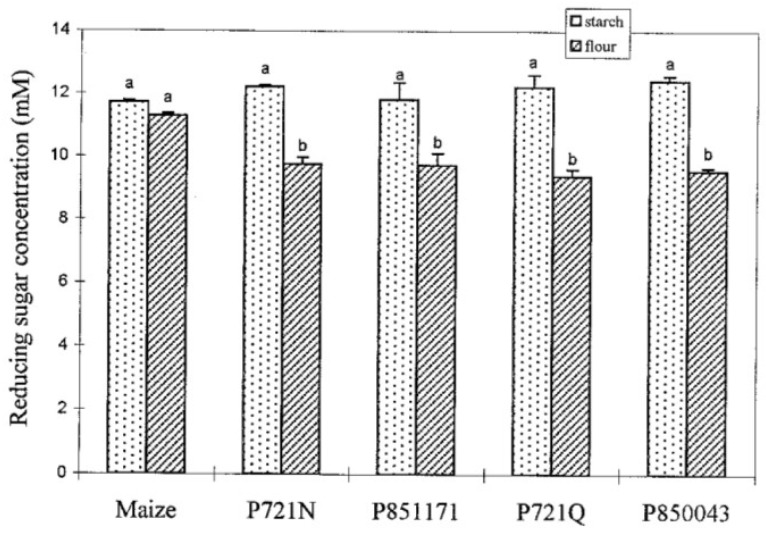
Starch digestibility of cooked isolated starch and flour samples after 2 h of α-amylase hydrolysis. ^ab^ Means not containing a common letter are significantly (*p* < 0.05) different. P721N, P851171, P721Q and P850043 are four cultivars of sorghum that represent different types of endosperms [118]. Figure 6. Used with permission of John Wiley & Sons from Low α-amylase starch digestibility of cooked sorghum flours and the effect of protein, Cereal Chemistry, vol. 75, pp. 710–713, Zhang & Hamaker, [118], published on behalf of the Cereals & Grains Association.

Sorghum and maize vary in how cooking their flour or isolated starch affects their starch digestibility and gelatinization. Starch digestibility in cooked maize and sorghum flours by α-amylase showed similar trends as their protein digestibility that was presented in [167]. Cooked sorghum flour had a significantly lower starch digestibility than cooked maize flour (Figure 6). Interestingly, digestibility of isolated sorghum starch was similar to digestibility of isolated maize starch (Figure 6) [118]. Cooked isolated sorghum starch had higher α-amylase digestibility (70.25%) than uncooked isolated sorghum starch (55.40%) and higher glucoamylase digestibility (64%) than uncooked isolated sorghum starch (12.5%) [35]. The same enzymatic treatment was used to study the digestibility of starch in isolated starch and flours of sorghum and maize (Figure 6). Hence, the difference in the digestibility of cooked sorghum starch versus cooked sorghum flour was due to non-starch flour components [118] such as proteins, lipids, dietary fiber and/or anti-nutritional factors [213,214]. In isolated starch, gelatinization is influenced by the botanical origin, granule size and shape, amylose and amylopectin content, and degree of crystallinity [215]. In flour, the gelatinization of starch is affected by the flour particle size [216]. The low digestibility of sorghum starch is associated with the low digestibility of sorghum protein [118]. The grain structure, protein composition, and starch granule structure of sorghum are very similar to maize. However, maize protein did not affect the digestibility of maize starch upon cooking. The starch digestibility of cooked isolated maize starch and cooked maize flour were not significantly different as depicted in Figure 6 [118]. Hence, examining the microstructure will explain why proteins and anti-nutritional factors in sorghum are hindering the digestibility of sorghum starch when cooked.

### 7.4. Microstructural Changes upon Heating

A study was conducted to understand the changes occurring to the microstructure of sorghum upon heating to further understand why the behavior of sorghum protein differs from other cereal proteins. Cooking changes the microstructure of sorghum protein. Extended web-like or fibril-like, and rigid sheet-like structures were formed when sorghum flour was cooked [217]. These structures form due to enhanced intermolecular interactions among proteins, which include formation of hydrogen bonds, hydrophobic interactions and/or disulfide bonds. Compared to the web-like fibril structure, the sheet-like structure contains higher concentration of protein bodies surrounded by the protein matrix. The web-like microstructure was also observed in maize flour that was cooked the same way. In maize, however, the web-like protein matrix seemed to have collapsed shortly after formation. Maize and sorghum prolamins are very similar in their amino acid composition [218]. However, the difference in the structural changes in their proteins during cooking may further affect their difference in digestibility. These resilient web-like and sheet-like structures may entrap the gelatinized starch and reduce the accessibility of enzymes from reaching the starch, resulting in poor digestibility of starch. Due to the collapse of the web-like protein matrix in maize, the gelatinized starch may not be encapsulated in the protein matrix and hence is exposed to starch-degrading enzymes. The protein microstructure formed during the cooking of sorghum acts as a barrier between the starch and α-amylase resulting in lower starch digestibility [117,219].

## 8. Treatments to Alter Digestibility of Starch and Protein

### 8.1. General Protein Modification

Since it is widely understood that the low digestibility of sorghum starch is highly associated with its protein, many studies have specifically focused on increasing the in vitro sorghum protein digestibility to directly enhance starch digestibility. There are many types of modifications of plant-based proteins that can be performed to improve nutritional properties. These include various physical, chemical, biological, and other approaches [220]. Physical approaches include heating, gamma irradiation, electron beam irradiation, ultraviolet radiation, pulsed-electric field, high pressure treatment, sonication, extrusion, ball mill treatment, cold atmospheric plasma processing, and ultrafiltration. Chemical modifications include glycation, phosphorylation, acylation, deamidation, cationization, and pH adjustment. Biological treatments include enzymatic and fermentation. Other modifications include complexation and amyloid fibrillization. Several of these modifications and other modifications have been applied to attempt to improve sorghum starch and protein digestibility [220].

### 8.2. Dehulling and Decortication

Dehulling and decortication can improve sorghum digestibility. Dehulling raw sorghum was generally found to increase in vitro protein digestibility in Dutch sorghum varieties by removing phenolic compounds to avoid possible protein-phenolic interactions [98]. Decortication of sorghum grain involves removal of the pericarp and testa layer (if present) using mechanical abrasion. Removal of these two components of grains leaves behind the starch and protein containing endosperm. Decortication of the grain removes non-starch components resulting in increased starch content as a percentage of remaining grain weight [61]. The apparent protein quality and digestibility of sorghum can be improved by decortication and extrusion processing [221].

### 8.3. Extrusion

Extrusion is a food processing method whereby moistened, starch and/or protein rich materials are cooked and formed into a viscous, plastic-like dough [222]. Raw materials are subjected to high temperatures with high shear and high pressure within the extruder. Starch is gelatinized before being pushed through a die in extrusion cooking. A die provides a small opening for the extruded product (extrudates) to exit. Upon exiting, the product rapidly expands due to a sudden vapor pressure decrease as the moisture is flashed off as steam [223].

Changes in starch and fiber contents after extrusion have been reported. High shear forces during extrusion can physically cleave glycosidic linkages in starch to form lower molecular weight fragments, and extent of fragmentation can vary with the molecular weight of the starch [224]. Fang et al. [225] have reported contents of 73.26% for total starch and 23.59% for amylose in unprocessed sorghum whole grain and contents of 80.35% for total starch and 31.66% for amylose in extruded sorghum whole grain based on a dry weight basis. Total dietary fiber, insoluble dietary fiber, and soluble dietary fiber contents of sorghum based upon a dry weight basis increased from 1.86%, 1.31%, and 0.55%, respectively, to 5.43%, 4.39%, and 0.94%, respectively, upon extrusion [225].

Extrusion improves sorghum digestibility. Llopart et al. [226] found that protein digestibility of whole red sorghum flours containing 14% moisture increased from 53.18% to 70.02% when extruded at 182 °C. Li and Sopade [227] reported that the rate of protein digestion of a multigrain blend of 60% sorghum and 40% barley was increased by extruding with moisture contents greater than 30% and temperatures greater than 140 °C. A method combining extrusion with an α-amylase treatment for starch liquefaction was developed to concentrate proteins from sorghum flour, and in vitro protein digestibilities up to 66% were measured for samples processed by this procedure [228].

Sorghum cooked with extrusion has a higher digestibility than sorghum traditionally cooked with water and heat. Extrusion cooking improved sorghum protein digestibility from 44.8% to 74.6% for the CS 3541 variety and from 43.3% to 68.2% for the US Market class [166]. However, these protein digestibility values for the extrusion cooked samples from the Fapojuwo et al. [166] study were actually less than the protein digestibility of uncooked sorghum (80.7%) [167]. It must also be noted that these data be viewed with caution, as experiments were conducted as parts of different studies. The difference in hydrolysis is due to the methodology used in these studies. Fapojuwo et al. [166] treated raw sorghum and sorghum extrudates with boiling water for 5 min to prepare a thin porridge before measuring the protein digestibility. Hence, the protein digestibility of this raw sorghum is actually reflecting the protein digestibility of wet cooked sorghum. This value is closer to the cooked protein digestibility (74.6% and 68.2%).

Mechanical force and heat from extrusion can break the disulfide bonds of γ-kafirin at the periphery of the protein body [229]. Although isolated α-kafirin is highly digestible, α-kafirin is surrounded by the highly cross-linked γ-kafirin, which barricades the starch making it inaccessible to digestive enzymes [230]. The heat generated from traditional cooking of sorghum is not strong enough to break the disulfide bonds.

The amylose and moisture content of sorghum can affect the properties of extruded sorghum. Sorghum with a low amylose content that was extruded at a lower moisture content (17.6%) had higher expansion, lower density, thinner air cell walls, and higher water solubility [61] than sorghum extruded at a high moisture content. The latter sorghum was found to have intact starch granules with retained birefringence, an indication of incomplete gelatinization. Extrudates from wx sorghum were an appropriate matrix for extruded snacks. The same study also reported a higher sensory acceptability of thin porridges prepared from extrusion products from heterowaxy sorghum than from nonwaxy sorghum extruded products [61]. Additional research results about protein and starch digestibility from extrusion of sorghum and millets can be found in Bhattarai et al. [231].

To maximize starch digestion for animal feed, Mahasukhonthachat et al. [116] recommended extruding sorghum at medium moisture (30%) and at 250 rpm. Conversely, to improve consumer acceptability of directly expanded sorghum extrudates in human nutrition, Mahasukhonthachat et al. [116] recommended extruding sorghum at medium moisture (30%) and at 150 rpm to minimize starch digestion leading to moderately high transverse expansion, bulk density, initial RVA viscosity, and water absorption indice.

### 8.4. Steam Explosion Treatments

Steam explosion technology is a promising pretreatment that can break walls for improving extraction rates in a wide variety of plant-based and animal-based by-products [232]. RDS decreased from 35.93% for untreated native sorghum flour to 23.00% after steam explosion at 1.2 MPa for 180 s, and RS increased from 39.89% to 54.08% after this steam explosion treatment [233]. Ma et al. [234] reported that the anti-digestion of low GI foods can be improved by enhancing glucose-binding capacity of sorghum total dietary fiber resulting from physical and structural changes and by inhibiting α-amylase activity by the total dietary fiber caused by steam explosion treatments.

### 8.5. Popping

Popping is an explosion of starch granules to yield a foam-like structure that disrupts starch–protein matrices present in the grain. NMR analysis of sorghum protein revealed that changes in the conformation of the secondary structure of protein are due to heat denaturation and aggregation. The analysis showed an increased β-sheet character in popping, but to a lesser extent than that observed for wet cooking [212]. This explains why popping sorghum, as opposed to wet cooking, leads to higher protein and starch digestibility and a reduced resistant starch content [34]. Popped sorghum had a protein digestibility of 73.5% versus 64.8% for wet cooked sorghum. Popping sorghum also completely inactivates α-amylase inhibitors [200].

### 8.6. Steam-Flaking and Micronization

Steam-flaking is achieved by steaming grains at atmospheric pressure followed by flaking through a set of rollers. Tempering sorghum before steam-flaking increases the degree of gelatinization for higher hydration of starch and protein [235]. In steam-flaking, starch granules in sorghum grain swell extensively and gelatinize completely, and heat and moisture are distributed throughout the kernel when the grain is rolled. This could contribute to disruption of the protein matrix and liberation of gelatinized starch from the matrix. Hence, the starch becomes more readily available to digestive enzymes. Therefore, steam-flaked sorghum grains are highly susceptible to α-amylase and amyloglucosidase [127,236].

Micronization is a process in which a material such as sorghum is heat treated by infrared irradiation and then passed through an extruding type of roller mill. Materials are then finely ground to reduce particle size. Shiau and Yang [237] performed micronization of sorghum at 102 °C, 250 °C, and 282 °C and found that micronization performed at 250 °C resulted in the highest in vitro starch availability measured by gas generation.

### 8.7. Microwaving with or Without Infrared Energy

Microwave energy, with or without also applying infrared (IR) energy, can alter sorghum digestibility. Li et al. [156] found that a microwave treatment of 600 watts for 6 min on three sorghum cultivars lowered RDS content but raised SDS and RS contents. Baah et al. [195] reported lower estimated GI and RDS content but higher RS content of raw sorghum, sorghum with added non-tannin extract, and sorghum with added tannin extract after microwave heat moisture treatment. Also, in vitro protein digestibility of raw sorghum and sorghum with added non-tannin extract decreased with microwave heat moisture treatment in their study. In another study, Baah et al. [196] performed heat-moisture treatments on white non-tannin, red non-tannin, and red tannin sorghum meals (25% moisture) by IR and microwave energy at 250 W for 15 min in a hot air tunnel. They found that in vitro protein digestibility was highest for white non-tannin sorghum and lowest for red tannin sorghum, but the treatment effect was not significant for in vitro protein digestibility. Also, they found a higher percentage RS and lower percentage RDS for the red tannin sorghum than for the red non-tannin and white non-tannin sorghums, but the treatment effect for starch digestibility depended on the type of starch [196]. Wang et al. [38] found that microwave treatment of sorghum starch after partial amylose removal increased its RS content due to disruption of the structure.

### 8.8. E-Beam Irradiation

E-beam irradiation is yet another technique that can be used to improve starch digestibility. Jaiswal et al. [238] reported that irradiation with an electron beam dose of 5 kGy led to a decrease in RS content from 38.0% to 29.0% for starch isolated from Phule Maldandi (a non-pigmented (white) sorghum cultivar) and from 28.9% to 20.8% for starch isolated from Phule Rohini (a red pigmented sorghum cultivar). These decreases can be explained by decreased tannin and starch interactions and production of shorter chains after irradiation. Liang et al. [239] applied acid hydrolysis for 1 and 6 h with 2.2 M HCl and E-beam irradiation set at 2, 4, and 8 kGy by a 10 MeV/20 kW EBI generator to sorghum starch and found that acid hydrolysis and E-beam irradiation increased RDS content but decreased RS content compared to the native starch. Although E-beam irradiation did not appreciably change the starch granule surface and growth rings when viewed by scanning electron microscopy and confocal laser scanning microscopy, E-beam irradiation fragments and depolymerizes chains of starch molecules and breaks chemical bonds. Acid hydrolysis appeared to corrode and break starch granule surfaces, forming cracks and perforations. The hydrolytic area and enzyme binding sites are increased within this broken structure to increase the RDS content [239].

Recent research by Lin et al. [240] on E-beam irradiation of sorghum flour provides important insights into how this treatment affects the flour’s physicochemical properties and processing suitability. Their findings demonstrated that low-dose E-beam irradiation (4 kGy) improved the fermentation properties of sorghum-wheat mixed dough, optimized microstructure, and enhanced textural properties of the resulting bread. The E-beam treatment enhanced flour solubility by modifying component structures while reducing swelling power, viscosity, and thermal properties. This modification improves starch degradation, which promotes optimal dough rheological properties during fermentation. Importantly, they found that low-dose E-beam irradiation could reduce the destructive effects of sorghum on the gluten network in mixed doughs, suggesting this technology could significantly improve the processing suitability of sorghum flour in various cereal-based products.

### 8.9. Ohmic Heating

Ohmic heating is an electric field-based technology that can provide uniform and fast heating for quicker processing time [241]. Flores-García et al. [242] subjected two sorghum genotypes with different amylose contents to ohmic heating at various electric field strengths and found that resistant starch content increased with increasing electric field strengths between 0 (native starch) and 70 V/cm.

### 8.10. Cold Plasma Treatment

Cold plasma technology is a relatively new non-thermal treatment that forms high-energy reactive species through ionized gases and can be used for decontaminating food, improving food quality, degrading toxins, and modifying packaging material surfaces [243]. Zhou et al. [244] applied a dielectric barrier discharge cold plasma pretreatment to sorghum and used scanning electron micrroscopy to observe a rougher and more textured seed surface with surface etching (pitting and minor erosion) compared to the untreated sorghum seed coat. This etching could lead to initiation sites for starch hydrolysis [244]. Gao et al. [245] reported that a dielectric barrier discharge plasma treatment (20 kV and 1 kHz frequency for 30 s with a 2 mm discharge distance) increased the RDS of sorghum starch from 17.21% to 25.52% and the SDS from 10.65% to 11.16% but decreased the RS from 72.14% to 63.32% compared to native sorghum starch.

### 8.11. Supercritical Carbon Dioxide Drying

Supercritical carbon dioxide drying is a drying procedure which uses carbon dioxide above its critical temperature and pressure to help material retain a delicate microstructure. Protein extracted from defatted sorghum flour subjected to supercritical carbon dioxide drying had a significantly higher digestibility (47.1%) than protein extract subjected to freeze drying (33.7%) [246]. These authors explain these differences in digestibilities by the dense structure and larger particle sizes for the freeze-dried samples that impede digestion.

### 8.12. Ozone Treatment

Ozone treatments can be used as a chemical method to inactivate microorganisms in a wide variety of foods [247]. Palavecino et al. [248] found that ozonation increased protein digestibility but decreased rate of starch hydrolysis in extruded sorghum flour. Thakur et al. [249] reported that in vitro starch digestibility of soup mixes containing sorghum starches increased from 29.95% for native sorghum starch to 32.45% for ozonated sorghum starch.

### 8.13. Milling

Various types of milling are used to reduce sorghum particle size. Dhanya et al. [102] obtained smaller particle sizes in sorghum flour samples milled using a hammer mill (geometric means of 64.26 μm for raw and 108.93 μm for microwave parboiled white sorghum) compared to burr and ball mills. The type of milling before extrusion affects in vitro starch digestibility as digestion occurred faster after cryo milling than in hammer milling [250]. Extrusion also led to a higher starch digestibility in their experiment. Although various feed rates and temperatures did not significantly affect the in vitro starch digestibility properties of a sorghum–barley (60:40) blend, moisture level and screw speed affected certain in vitro starch digestibility properties [250]. Hammer-milling can lead to small changes in the grain particle structure probably due to additional frictional heat and mechanical effects that can affect starch digestion kinetics [111]. For example, the starch from hammer-milled samples with a volume-weighted mean particle size of 256 μm generally digested more readily than starch from cryo-milled samples with a volume-weighted mean particle size of 119 μm [111]. Al-Rabadi et al. [131] passed hammer-milled sorghum through a series of seven analytical sieves into a pan with a sieve shaker and found that smaller particles were digested faster than larger particles due to increased surface area for enzyme access. Likewise, Xu et al. [206] found that starch digestibility was inversely related to sorghum flour particle size. Furthermore, the extent of digestion of non-fractionated sorghum starch after a given incubation time can be predicted based upon the weighted digestibility of each particle size fraction, allowing an approximate particle size fraction to be produced [131]. However, variations in mechanical properties such as endosperm hardness may alter particle size distributions [131]. However, Xu et al. [206] found that endosperm hardness did not clearly affect rate of starch digestion in sorghum flour.

### 8.14. High Pressure Treatments

High hydrostatic pressure treatments affect starch digestibility. Liu et al. [62] measured RDS, SDS, and RS of control sorghum starch and sorghum starch subjected to hydrostatic pressures between 120 MPa and 600 MPa in increments of 120 MPa for 20 min at room temperature. RDS, SDS, and RS values at 600 MPa in their study were 20.3%, 45.1%, and 13.4%, respectively. They found that RDS decreased but SDS and RS increased with increasing hydrostatic pressures. Likewise, they found increasing amylose content but decreasing amylopectin content with increasing hydrostatic pressure [62]. The increase in the amylose content may be explained by limited amylose leaching arising from amylose-amylose, amylose-amylopectin, and amylose–lipid interaction during high hydrostatic pressure processing. Also, amylopectin may degrade during high hydrostatic pressure processing [62]. Oliveira et al. [251] applied high hydrostatic pressure processing of white sorghum starch at various holding temperatures and found decreased SDS but increased RS in the processed samples compared to the native sample. These changes in digestibility can be explained by structural alterations from rearrangement and recombination of leached amylose with naturally present lipids forming a more compact structure that is less accessible to slower digestion without major changes to rapid digestion [251].

High hydrostatic pressure treatments also affect protein digestibility. Correia et al. [207] measured in vitro protein digestibility of cooked and uncooked sorghum samples both with and without an applied high hydrostatic pressure treatment of 300 MPa for 15 min. Although high pressure treatment had a relatively small effect on protein digestibility of uncooked sorghum (42.1% digestibility before pressure treatment versus 43.5% digestibility after pressure treatment), applying high pressure treatment to cooked sorghum (with 16.1% digestibility) increased the digestibility to 25.4% if applied after cooking or to 35.3% if applied before cooking [207]. Also, Correia et al. [207] reported that digestibility decreases with increasing applied pressure (from 27.6% digestibility at 100 MPa to 24.3% digestibility at 450 MPa for 15 min) and with increasing time (36.0% digestibility for 5 min to 28.7% digestibility for 30 min at 300 MPa). Although protein unfolding and disulfide bond disruption allowing pepsin access to the proteins may occur up to a certain pressure, subsequent aggregation and re-association due to hydrophobic interactions occur at higher pressures to decrease protein digestibility [207].

High-pressure homogenization has been applied to sorghum suspensions. The average size of particles in a sorghum suspension was reduced from 9-66 μm to 1.8–2.5 μm upon high-pressure homogenization, enhancing protein dispersibility [83]. High-pressure treatment of sorghum batter has the potential to improve the quality of sorghum-based gluten-free products. A problem for food applications of sorghum proteins can arise from its low water solubility, but high-pressure homogenization can alter the size and structural organization of protein-based aggregates to assist their dispersibility in aqueous media [83]. These changes may affect sorghum digestibility.

### 8.15. Ultrasonication

Ultrasonication (or sonication) is another technique that can be used to alter properties of kafirin and starch including improved digestibility. Sharma et al. [252] showed that in vitro starch digestibility can be improved from 30.63% to 50.29% by treating sorghum starch with ultrasonication at 30% amplitude for 20 min. This increased digestibility from ultrasonication can be explained by formation of smaller starch granule sizes, indentations, holes, and cracks and degradation of forces within and between starch granules as shown by scanning electron microscopy. Although native sorghum starch granules were irregular in shape, their surfaces were smooth with no evidence of fissures [252]. The ultrasound treatment of sorghum flour slurries results in the release of bound starch by disrupting the protein matrix that encases starch granules and amylose–lipid complexes [253]. Generation of shear forces during sonication disrupts the hydrophobic interactions or the inter-molecular disulfide bonds in proteins [254]. Sonication also changes surface hydrophobicity and secondary structures of protein [255]. Sullivan et al. [256] used ultrasonication to improve protein digestibility of sorghum flour by altering the secondary structure of kafirin as shown by changes in spectra of circular dichroism and Fourier-transform infrared spectroscopy.

This method is generally used to release maximum glucose from starch for bioethanol production. The enzymatic treatment of starch for bioethanol production includes usage of both highly thermostable α-amylase and commercial amyloglucosidase, similar to those which are used in the Englyst in vitro starch digestibility assays [144]. Disrupting the protein matrix increases the availability of more starch molecules to enzymatic hydrolysis and results in increased yields of up to 90% [253].

### 8.16. Reducing Agents

Reducing agents can improve the in vitro sorghum prolamin digestibility since they can break the disulfide bonds in the protein matrix that were formed during cooking of sorghum. Like for the improvement in the digestibility from ultrasound treatment, reducing agents also disrupt the protein microstructure to allow enzymes to reach the starch molecules that were previously impenetrable. The reducing agent dithiothreitol was the most effective, followed by bisulfite, 2-mercaptoethanol, and L-cysteine, up to 100 mM concentration, and it enhances sorghum protein digestibility up to 81.8% [208]. Once the cross-linked proteins are disentangled by the reducing agent, the entrapped starch easily becomes accessible for enzymatic reaction.

### 8.17. Enzyme Treatment, Ingredient Fortification, and Fermentation

Enzyme treatment and ingredient fortification can be used to change sorghum digestibility. Pepsin treatment of cooked sorghum flour decreased the percentage of RS from 16.93 to 23.99% without pepsin treatment to 4.86–12.53% with pepsin treatment and performing this pepsin treatment at pH 2.0 rather than at pH 1.3 resulted in greater protein and starch digestion [206]. Liu et al. [257] found that in vitro starch digestibility of cooked sorghum noodles decreased upon addition of wheat protein, whey protein isolate, and egg white protein due to formation of additional starch–protein and protein–protein interactions. They found that both RDS and SDS decreased but RS increased with increasing added wheat protein level from 0% (control) to 5%. A decrease in RDS and an increase in RS were found with increased added whey protein isolate level and egg white protein level from 0% (control) to 3%, but an increase in RDS and a decrease in RS occurred upon 3% to 5% addition. Adeyanju et al. [258] increased the in vitro protein digestibility of fermented and unfermented flours and porridges from sorghum by fortifying with okara flour.

Fermentation either with or without additional fortification can be used to improve sorghum digestibility. Day and Morawicki [259] increased protein digestibility by fermenting sorghum with baker’s yeast (*Saccharomyces cerevisiae*) and the amylolytic yeast *Lipomyces kononenkoae* compared to thermally processed unfermented control samples. Ogodo et al. [260] measured in vitro starch digestibility and in vitro protein digestibility of sorghum flour naturally fermented, fermented using a lactic acid bacteria previously isolated from fermenting maize, and fermented using a lactic acid bacteria previously isolated from fermenting sorghum and found higher digestibilities from the sorghum fermented with either maize or sorghum consortia. Moriconi et al. [261] combined fermented sorghum flour with germinated bottle gourd seed flour. They found highest in vitro starch digestibility for fermented sorghum porridge and highest in vitro protein digestibility in blended sorghum porridge with 48 h fermentation with non-germinated seed flour. Also, Moriconi et al. [262] found that in vitro starch digestibility increased when co-fermenting sorghum porridge fortified with baobab pulp compared to performing fermentation without baobab pulp to be used as an infant complementary food. Mustafa et al. [263] fermented and then cooked a meal of 100% sorghum flour and meals of 10, 15, or 20% defatted pumpkin seed pulp and 20, 15, or 10% wheat flour, respectively, incorporated into 70% sorghum flour. They found that the uncooked fermented meals had the highest in vitro protein digestibilities, but the cooked meals retained higher digestibilities than the raw meals. The increased digestibilities resulting from fermentation can likely be explained by enhanced peptidase activity, activated endogenous proteases, enhanced protein solubility at lower pH values, and degradation of antinutrients (tannins, phytates, trypsin inhibitors, and polyphenols) during fermentation.

### 8.18. Seed Germination and Malting

Seed germination is another way to improve the nutritional value of sorghum. Afify et al. [264] found that in vitro protein digestibilities of three white sorghum varieties ranged from 50.94 to 52.09% when measured before germination but significantly increased after germination treatments. Protein digestibility was improved by germination for both raw and cooked sorghum, especially for tannin-poor genotypes [265]. Proteases and amylases are activated by germination [266]. When examined by scanning electron microscopy, germination led to starch granule pitting and loosening of the protein–starch matrix [265]. Abdelbost et al. [265] hypothesized that germination assists with kafirin unfolding by reducing intramolecular disulfide bonding and by proteolytic cleavage. Likewise, Wu et al. [267] found that in vitro digestibility (both RDS and SDS) also increased for red sorghum starch, and this increase was explained by an enzymatic loosening of the microstructure of starch granules during germination. Starch granule hydrolysis from outer layers to inner layers occurs as enzymes can more readily bind the substrate as shown by scanning electron microscopy [267].

Seed germination combined with extrusion can also improve the nutritional value of sorghum. Madrigales et al. [168] applied a germination process (37 °C for 69 h) and an extrusion process (137 °C at 134 rpm) to whole sorghum grain and found increases for in vitro protein digestibility of 10% from the germination process and 13% from the extrusion process. The hypoglycemic potential was improved when applying the germination process and the extrusion process. The latter can be attributed to the release of phenolic compounds that can bind to the active site of α-amylase to modify its enzymatic activity. Also, Maillard reaction products with hypoglycemic potential can form during the extrusion process [168].

Malt pretreatment can improve in vitro protein digestibility of sorghum flour [193]. These authors germinated sorghum seeds for up to three days to obtain sorghum malts and added them at concentrations up to 10% to the sorghum flour. After shaking, mixing with water, and incubating for up to 120 min in a shaker, the samples were dried and ground. The in vitro protein digestibility progressively increased and the phytic acid content progressively decreased with increasing days of malting, increasing concentration added to the sorghum flour, and increasing incubation time. The increase in the in vitro protein digestibility was probably due to indigenous protease activity from the sprouted seeds.

### 8.19. Summary of Methods

Sorghum digestibility can be improved by various processing techniques including dehulling, decortication, extrusion, popping, steam flaking, E-beam irradiation, ohmic heating, cold plasma treatment, supercritical carbon dioxide drying, ultrasonication, addition of reducing agents, pepsin treatment, fermentation, germination, and malting. Many of the processing methods presented here are similar in how they improve the digestibility of sorghum starch and protein. These treatments disrupt the protein–starch matrix of sorghum to release the bound starches to increase its accessibility to digestive enzymes. These processing methods can provide sufficient energy to break the resistant protein–starch matrix of sorghum. These treatments can also inactivate the amylase and trypsin inhibitors that are naturally found in sorghum since these enzyme inhibitors are heat-labile and can be inactivated by thermal processing [199,203] and fermentation [268]. The inactivation of amylase and trypsin inhibitors may further improve sorghum digestibility.

## 9. Uses of Sorghum in Baking

Sorghum flour is currently used in a variety of gluten-free baked goods. It provides a bland flavor and acceptable texture. Because of the organization of kafirin subclasses discussed previously, it has generally been accepted that kafirin in its native state has little effect on the quality of baked goods. However, recent research by Rumler et al. [269] has demonstrated that the sorghum variety plays a significant role in the final quality of bakery products. Their study examining eight different sorghum varieties revealed significant differences in rheological properties, baking performance, and sensory acceptance of sorghum-wheat composite breads. Disruption of kafirin protein bodies within sorghum flour can improve sorghum-based gluten-free bread quality. Schober et al. [270] used a sourdough process to digest native kafirin in flour by *Lactiplantibacillus* (formerly *Lactobacillus*) *plantarum*. Size exclusion chromatography (SEC) showed that there was a decrease in the molecular weight of the extractable kafirin. Bread baked from the fermented flour had improved crumb structure, loaf volume, and rounding of the top loaf when compared to breads made from unmodified control flour. Laser scanning confocal microscopy of the bread crumb structure made from the fermented flour showed little to no aggregated proteins, as opposed to the control. This indicated increased solubility of sorghum endosperm matrix proteins. Schober et al. [270] claimed that degradation of sorghum proteins via sourdough fermentation would prevent interference of starch gel formation, where protein aggregation upon heating was found to weaken starch gels. This in turn would allow for greater gas retention by not allowing native kafirin to disrupt gas cells within the bread crumb starch matrix. Although thorough, the focus of this work was not digestibility.

It would be interesting to address the effects of *Lactiplantibacillus* (formerly *Lactobacillus*) *plantarum* fermentation on the carbohydrate and protein digestibility in sorghum. Some strains of *Lactobacillus* have been found to have amylolytic effects [271], which could also influence digestibility in the human gut.

## 10. Effects on Gut Microbiome

While the previous sections discussed sorghum starch and proteins, endosperm structure, starch and protein digestibility, overall health benefits, anti-nutritional factors, cooking and other processing treatments, and baking, this next section will examine specific effects of sorghum consumption on the gut microbiome. The gut microbiome includes all the microorganisms (bacteria, archaea, fungi, protozoa, and viruses including their genes and their products) that reside within the large intestine and play an important role in health and disease [272]. Changes in diet can affect the composition of the gut microbiome, and changes in the gut microbiome affect an individual’s health [273].

Consumption of various types of starch, gluten, sorghum, and condensed tannins in sorghum affects the gut microbiome. RS is not absorbed within the intestines, leading to significant production of short-chain fatty acids and modulation of gut-associated immunity and gut microbiota. Therefore, RS can be considered as a prebiotic [157]. Ze et al. [274] found that *Ruminococcus bromii* plays an important role for degrading RS in the large intestine. Compared to a high-gluten diet, a low-gluten diet leads to decreases in four species of *Bifidobacteria* spp., two species of *Dorea*, *Blautia wexlerae*, two species of the *Lachnospiraceae* family, the butyrate-producing bacteria *Anaeostipes hadrus* and *Eubacterium hallii*, and some unclassified species in the intestinal microbiome [275]. Bonder et al. [276] reported a decrease in *Veillonellaceae* (a pro-inflammatory family of bacteria found in increasing amounts in patients with IBD, IBS, and cirrhosis) upon adoption of a gluten-free diet.

Recent studies have specifically investigated the impact of sorghum consumption on the gut microbiome. Martinez et al. [277] fed a group of *Wistar* rats an unsupplemented high-fat high-fructose diet and another group of rats a high-fat high-fructose diet supplemented with sorghum flour BRS 305 hybrid and found that the rats in this sorghum group had a higher *Firmicutes*/*Bacteroidetes* ratio and increased abundance of *Roseburia* and *Lachnospiraceae_NK4A136_group* (a potential probiotic) within their intestinal microbiota compared to the rats fed the unsupplemented high-fat high-fructose diet. Feeding extruded sorghum flour to rats that were fed a hyperlipid diet increased their *Bacteroidetes* and decreased their *Firmicutes*, inflammation, and oxidative stress in their gut [278].

Clinical evidence supporting sorghum’s beneficial effects on human gut microbiota has also emerged. Upon consumption of the same genotype (SC 319) of extruded sorghum by overweight men, Lúcio et al. [279] found reduction in *Clostridium sensu stricto 1* (a cluster of commensal and pathogenic species), *Dorea* (positively associated with prediabetes and glucose concentrations), and *Odoribacter* (often associated with expression of inflammatory cytokines) in stool samples and reported improved body composition and weight loss compared to a control wheat group, revealing prebiotic potential of this sorghum.

The bioactive components in sorghum appear to significantly influence its effects on gut microbiota. Yang et al. [280] showed that condensed tannins in sorghum stimulate the growth of *Faecalibacterium prausnitzii*, which implies that sorghum consumption may be especially beneficial for individuals with inflammatory bowel diseases. This finding is particularly significant as *F. prausnitzii* is one of the most abundant butyrate-producing bacteria in the human intestinal microbiota and is known to have anti-inflammatory properties [281].

Altering the type of starch in sorghum affects the gut microbiome after consumption. Waxy starches result from mutations in which amylose biosynthesis is blocked, resulting in greater than 90% of the starch being amylopectin in the cereal grains. Although waxy starches produce desirable properties in certain situations such as production of sticky rice, it can lead to undesirable changes in the gut microbiome [282]. They performed in vitro fermentation of near-isogenic lines of wild type sorghum and waxy sorghum grain and other cereal grains using the microbiome from human stool. The wild type phenotypes had 7 to 13 times more amylose and 3 to 5 times more resistant starch than the waxy phenotypes. Fermentation of waxy lines led to an increase in the *Proteobacteria* (*Pseudomonadota*) and *Bacteroidetes* (*Bacteroidota*) phyla, while fermentation of wild type lines led to an increase in bacteria from the *Firmicutes* (*Bacillota*) and *Actinobacteria* (*Actinomycetota*) phyla. These effects were tested in germ-free mice inoculated with a donor human gut microbiota and found that mice fed waxy diets had increased weight gains compared to mice fed wild-type diets [282]. Processing methods applied to sorghum may also influence its effects on gut microbiota. Extrusion, fermentation, and germination not only affect sorghum digestibility as discussed in previous sections but may also alter the profile of bioactive compounds that reach the colon and interact with gut microbiota [283].

In summary, many potential factors related to sorghum can affect the gut microbiome. More research needs to be conducted to determine the effects of sorghum composition and processing on the composition and activity of the gut microbiome and resulting effects on host health. Future research should focus on identifying specific structure-function relationships between sorghum components and beneficial gut bacteria, and on elucidating the mechanisms by which sorghum consumption influences host metabolism and immunity through gut microbiota modulation.

## 11. Conclusions

Sorghum consumption plays an important role in the diets of a wide variety of populations and provides numerous health benefits. Diabetics can benefit from sorghum consumption because of the low starch digestibility. People who live in semi-arid climates often consume sorghum as a staple food, and celiac patients can consume sorghum-based foods as it does not contain gluten. These latter two groups could often benefit from an increased caloric intake by increasing starch and protein digestibility of sorghum. The digestibility of sorghum is less than many other cereals and is often further decreased upon cooking.

This review has comprehensively examined the factors influencing sorghum digestibility, including the unique structural features of sorghum starch and protein, the formation of resistant starch, and the role of anti-nutritional factors. We have highlighted how the complex interactions between sorghum kafirin proteins and starch, particularly during thermal processing, create a distinctive digestibility profile that differentiates sorghum from other cereal grains.

Various processing methods applied to sorghum to alter sorghum digestibility can help to meet these varied needs. Among these, extrusion, fermentation, germination, and E-beam irradiation have shown promise for improving sorghum protein and starch digestibility while maintaining beneficial bioactive compounds. The most effective approaches appear to be those that disrupt the protein–starch matrix formed during cooking while minimizing the formation of disulfide bonds between kafirin proteins.

Furthermore, factors affecting sorghum starch and protein digestibility may also affect the gut microbiome, so multiple factors must be considered for determining the effect of sorghum composition and processing on a person’s health. The emerging evidence suggesting that sorghum consumption may beneficially modulate the gut microbiota composition adds another dimension to its potential health benefits, particularly for individuals with metabolic or inflammatory conditions. Recent advances in understanding sorghum variety-specific effects on digestibility, baking quality, and sensory properties underscore the importance of genotype selection for specific food applications. The identification of varieties with favorable processing characteristics and consumer acceptance is crucial for expanding sorghum utilization in both traditional and novel food products. Therefore, many future research and product development opportunities exist for maximizing the health benefits provided by sorghum in our diets. Key research priorities should include (1) further elucidation of the molecular mechanisms underlying sorghum’s reduced digestibility upon cooking; (2) optimization of processing methods specific to different sorghum varieties; (3) development of improved breeding strategies to enhance both digestibility and bioactive compound profiles; and (4) clinical studies to validate the health benefits of optimally processed sorghum products, particularly regarding glycemic response, satiety, and gut health. Through these concerted research efforts, sorghum’s full potential as a nutritious, sustainable, and health-promoting grain can be realized.

## Figures and Tables

**Table 1:** Summary of some starch, amylose, and amylopectin contents of sorghum provided in the literature.

Total Starch	Amylose	Amylopectin
57 to 69% (ungerminated) 55 to 64% (germinated) [30]	10 to 17% (ungerminated) 9 to 16% (germinated) [30]	45 to 54% (ungerminated) 43 to 52% (germinated) [30]
71.4% (normal) 71.2% (heterowaxy) 68.4% (waxy) [27]	23.7% (normal) 14.0% (heterowaxy) 0.0% (waxy) [27]	33.93–90.05% (Review by [31])
64.50 to 82.17% [32]	24–33% (in 95 Zimbabwean sorghum landraces) [33]	
73.5 to 76.3% (native) 74.3 to 78.3% (popped) [34]	0.6–32.69% (Review by [31])	
64.5% [35]	55.8% [36]	
55.6 to 75.2% (69.5% mean) [37]	23.42% for control [38]	
66.38% (white sorghum) 65.28% (red sorghum) [39]	32.99% [40]	
44.85% (whole sorghum flour) [41]	23.43 and 26.52% (sweet) 5.65 and 6.36% (waxy) [42]	
	7.42 to 36.44% [43]	
	22.2 to 24.7% (native) 17.4 to 21.5% (popped) [34]	
	21.2 to 30.2% (26.9% mean) [37]	

**Table 2:** Abundance of predicted type of secondary structures of sorghum flour protein, whole kafirin, and individual kafirins.

Protein	α-Helix	β-Sheet	β-Turn	Random Coil	Reference
	(%)	(%)	(%)	(%)	
Sorghum flour proteins	30–37	22–31	17–24	10–16	[83]
Whole kafirin	49	24	27		[79]
Whole kafirin	67.26–68.54	1.15–4.66	12.9	13.77	[80]
α_1_-kafirin	74		4	22	[82]
α_2_-kafirin	53		14	33	[82]
γ-kafirin	42		5	53	[82]
β-kafirin	64		13	23	[82]
δ-kafirin	41		14	45	[82]

**Table 3:** Predicted sizes of individual kafirins based upon protein modeling procedures.

Kafirin	Size (Å^3^)	Reference
α-	86.6 × 39.8 × 40.3	[80]
β-	66.7 × 46.0 × 30.4	[80]
γ-	52.6 × 48.6 × 35.8	[80]
δ-	32.6 × 39.0 × 42.0	[80]
α_1_-	48 × 72 × 42	[82]
α_2_-	45 × 86 × 40	[82]
β-	66 × 48 × 35	[82]
γ-	65 × 56 × 48	[82]
δ-	35 × 46 × 35	[82]

**Table 4:** Summary of some protein contents of sorghum provided in the literature.

Description of Samples	Protein Content	Reference
Described in review article	<0.51% to 14.50% range	[31]
Described in book	6% to 18% range (11% average)	[96]
15 sorghum genotypes	9.8% to 15.2% range (12.6% average)	[97]
Dutch sorghum whole grains	9.3% to 14.8% range	[98]
Sorghum accessions from Mali, Niger, Senegal, and Togo	Up to 18.32%	[99]
Sorghum lines and hybrids	10.3% to 16.8% range	[32]
Four cultivars of sorghum grain from Mozambique	7.35% to 9.39% range	[100]
Sorghum before malting	5.68%	[40]
Native and popped sorghum	9.7 to 9.8%	[34]
8 sorghum cultivars	6.8 to 19.6%	[101]
10,479 genotypes of sorghum grains	4.4 to 21.1% (11.4% average)	[37]
Parboiled, dried, and milled sorghum grains	6.35 to 8.65%	[102]
3 varieties of sorghum	8.7 to 9.1%	[103]
White sorghum Red sorghum	12.27% 12.59%	[39]
5 genotypes of sorghum whole grain flour	9.8% to 11.8%	[73]
Whole sorghum flour	11.67%	[41]
Native sorghum flour	4.17%	[104]

**Table 5:** RDS (rapidly digestible starch), SDS (slowly digestible starch), TDS (total digestible starch), and RS (resistant starch) of sorghum starch.

Type of Sample	RDS	SDS	TDS	RS	Reference
Four cultivars of sorghum grains from Mozambique	41.89 to 64.30%	18.98 to 28.93%	72.73 to 76.46% *	14.02 to 32.37%	[100]
Six varieties of red sorghum with varying amylose levels	89.89 to 93.11%	3.61 to 7.37%		2.56 to 3.39%	[152]
Normal sorghum starch Heterowaxy sorghum starch Waxy sorghum starch	12.5% 12.0% 21.5%	68.5% 61.7% 68.4%		17.9% 23.7% 8.4%	[27]
Control sorghum starch samples	59.71%	23.90%		16.50%	[38]
49 sorghum genotypes				0.31 to 65.65%	[154]
Pigmented sorghum White sorghum	19.4 to 30.6% 37.1%	34.1 to 40.8% 35.7%		4.2 to 21.4% 1.2%	[155]
Before microwaving 3 kinds of sorghum After microwaving 3 kinds of sorghum	31.10–33.78% to 23.38–27.90%		60.02–61.27% to 65.90–66.86%	5.09–7.62% to 6.12–9.76%	[156]

* TDS measured after 180 min of digestion.

**Table 6:** In vitro protein digestibility of various sorghum products.

Type of Sample	In Vitro Protein Digestibility	Reference
Dutch sorghum varieties (whole grain samples)	9 to 81%	[98]
Sorghum accessions from Mali, Niger, Senegal, and Togo	1 to 53%	[99]
Non-decorticated cooked sorghum Decorticated cooked sorghum Decorticated and extruded cooked sorghum	59.0 and 65.5% 56.8% 79.0%	[162]
Whole kernel uncooked sorghum meal Dehulled uncooked sorghum meal Whole kernel cooked sorghum meal Dehulled cooked sorghum meal (percent of sorghum protein solubilized by pepsin)	88.6 to 93.0% 78.2 to 85.7% 45.3 to 56.7% 37.1 to 43.0%	[103]
Fine red sorghum flour Fine white sorghum flour Red sorghum gluten meal White sorghum gluten meal	86.02% 86.38% 84.85% 85.30%	[164]
Seven Algerian sorghum cultivars	25 to 65%	[95]
Four sorghum cultivars from Mozambique	61.97 to 64.54%	[100]
Sorghum grains Sorghum protein concentrates Sorghum protein hydrolysates	13.18% 30.80% 47.14%	[165]
Native sorghum Popped sorghum	65.0 to 70.2% 67.9 to 73.5%	[34]
CS 3541—raw and added 4% Ca(OH)_2_, pH 11.0, 200 °C extrusion U.S. market class–raw and added 4% Ca(OH)_2_, pH 11.0, 200 °C extrusion	44.8 to 91.3% 43.3 to 83.5%	[166]
Cooked and uncooked sorghum	64.8 and 80.7%	[167]
2 h digestion of sorghum genotypes 3 h digestion of sorghum genotypes	36.64 to 79.51% 37.99 to 95.80%	[159]
Before germination-extrusion of flour After germination-extrusion of flour	58% 72%	[168]

## Data Availability

No new data were created or analyzed in this study. Data sharing is not applicable to this article.
